# VASN Enhances IGF2BP3 Stability via USP10 Deubiquitination to Promote Triple-negative Breast Cancer Paclitaxel Resistance

**DOI:** 10.7150/ijbs.136403

**Published:** 2026-07-13

**Authors:** Hao Liu, Zexiu Lu, Maoshan Chen, Zhenghang Li, Jian Chen, Xingyu Yang, Xueying Wan, Gang Tu, Manran Liu, Lingfeng Tang

**Affiliations:** 1 Department of Breast and Thyroid Surgery, Chongqing Key Laboratory of Molecular Oncology and Epigenetics, The First Affiliated Hospital of Chongqing Medical University, Chongqing, 400016, China.; 2 Key Laboratory of Laboratory Medical Diagnostics, Chinese Ministry of education, Chongqing Medical University, Chongqing 400016, China.

**Keywords:** VASN, chemoresistance, triple-negative breast cancer, ABCB1

## Abstract

Triple-negative breast cancer (TNBC) is an aggressive subtype lacking effective treatment options, and paclitaxel resistance remains a major clinical challenge. This study investigated the role of VASN in mediating paclitaxel resistance in TNBC by integrating transcriptomic and single-cell RNA sequencing data from public databases and clinical samples. Using paclitaxel-resistant TNBC cell lines (MDA-MB-231R and CAL-51R) and xenograft models, we revealed that VASN was significantly upregulated in resistant TNBC cells and tissues, correlating with poor prognosis. Mechanistically, CEBPB directly bound to the VASN promoter to activate its transcription, and VASN interacted with IGF2BP3 via its LRR domain, recruiting USP10 to deubiquitinate and stabilize IGF2BP3 by suppressing K48-linked polyubiquitination. Stabilized IGF2BP3 enhanced ABCB1 expression through m^6^A-dependent mRNA stabilization, activating the PI3K/AKT pathway and driving paclitaxel resistance. Genetic or pharmacological inhibition of VASN resensitized resistant cells to paclitaxel, and computational drug screening identified Trametinib as a candidate to downregulate VASN. Trametinib synergized with paclitaxel to effectively suppress tumor growth without obvious toxicity in resistant models. In summary, we uncovered a new CEBPB-VASN/IGF2BP3/USP10-ABCB1 axis responsible for paclitaxel resistance in TNBC. Targeting VASN with Trametinib in combination with paclitaxel represents a promising therapeutic strategy to overcome chemoresistance, offering a rationale for precision medicine in resistant TNBC.

## Introduction

Breast cancer (BC) is the leading malignancy in women globally, of which triple-negative breast cancer (TNBC) accounts for 15-20% of cases^1^. TNBC is characterized by high aggressiveness, elevated rates of recurrence and metastasis, and poor prognosis^2^. Given the absence of specific targets, taxane-based regimens remain a mainstay of TNBC treatment^3^. Although chemotherapy can provide clinical benefit to some TNBC patients, the development of chemoresistance significantly limits its therapeutic efficacy. Currently, only a subset of TNBC patients derive maximal benefit from chemotherapy^4^, ^5^. Notably, futile chemotherapy not only induces severe adverse events such as myelosuppression and cardiotoxicity, but also creates a heavy economic burden for patients and the healthcare system^6^.

VASN (vasorin) is consistently expressed from the embryonic stage through adulthood in various tissues, organs, and biological fluids^7^. Within the cardiovascular system, VASN is predominantly localized in the coronary arteries and aorta, and is present in both vascular smooth muscle cells and endothelial cells^8^. Initial research suggests its important role in vascular injury repair, with relatively enriched expression in the aorta, kidneys, and placental tissue^9^. Meanwhile, previous studies indicated that VASN acts as a tumor-promoting factor that drives the progression of multiple cancers^10^, ^11^. For instance, VASN promotes cancer cell proliferation, invasion, and tumorigenesis by activating the Notch1 signaling pathway in ARID1A-deficient lung adenocarcinoma^12^. Clinically, high VASN expression is significantly correlated with lung metastasis, advanced TNM stage, and poor prognosis. Furthermore, in glioma, VASN binds to VEGFR2, inducing its internalization and autophosphorylation, which subsequently activates the AKT pathway to promote angiogenesis^13^. Additionally, VASN functions as an oncogene in various other cancers, such as hepatocellular carcinoma, thyroid cancer, and gastric cancer, where it regulates proliferation, metastasis, angiogenesis, and chemoresistance^14^.

This study revealed that VASN expression is markedly upregulated in paclitaxel-resistant TNBC and drives the activation of a key pathway. Notably, we showed that suppressing VASN expression or pharmacological intervention was able to reverse paclitaxel resistance. Therefore, our findings indicate that VASN represents a druggable and specific therapeutic target for overcoming paclitaxel resistance in TNBC, offering a valuable reference for advancing precision medicine.

## Materials and methods

### Cell culture

The BC cell lines were purchased from the ATCC or DSMZ with detailed information provided in **Table S1**. Cells were cultured in DMEM or RPMI 1640 (Gibco, USA) with 10% FBS (HyClone). All cells cultures were incubated at 37℃ with 5% CO_2_. To ensure experimental reliability, all cell lines underwent authentication with matching rates exceeding 85% against the Cellosaurus database (v2024_01).

Both MDA-MB-231R and CAL-51R cells were established through long-term, stepwise exposure to paclitaxel. For all functional assays in this study, cells were cultured in paclitaxel-free medium for at least 48-72 hours prior to and during experimentation.

### Clinical specimens

For this study, tumor and matched adjacent normal specimens were collected from patients at the First Affiliated Hospital of Chongqing Medical University. And all cases were verified by pathological biopsy. Ethical approval was secured from our institutional committee (reference No. 2020-202), and obtained informed consent forms from all participants. The clinical pathological features and VASN expression analysis are shown in **Table S2**.

### The Cancer Genome Atlas (TCGA) and Gene Expression Omnibus (GEO) data collection

We extracted clinicogenomic datasets of invasive breast carcinoma from TCGA, which included HTSeq-FPKM normalized transcriptomic data and mRNA expression profiles. In this study, four mRNA expression datasets (GSE90564, GSE25066) were collected from the GEO repository (https://cancergenome.nih.gov) to investigate genes underlying paclitaxel resistance. Differential expression analysis of these datasets pinpointed consensus genes exhibiting significant expression alterations (|log₂FC| > 1, adjusted p < 0.05) when comparing resistant and sensitive phenotypes. Using the oncoPredict R package (v0.2), drug IC50 values were calculated for individual patients based on normalized FPKM data from TCGA-BRCA.

### ScRank-based drug sensitivity analysis

The GSE169246 dataset was retrieved from the GEO database, which comprises single-cell RNA sequencing data from tissues of 22 patients with advanced TNBC. The fastq files were aligned with the hg38 reference genome using the 10× Genomics Cell Ranger software to generate a gene expression matrix. Normalization and batch correction were subsequently applied. Next, cell clustering and visualization were conducted. Unsupervised clustering was employed to partition cells into subpopulations, which were annotated based on known marker genes. Subsequently, the scRank algorithm was executed using the preprocessed expression matrix and predefined gene sets associated with specific targets or drugs. This step computed a sensitivity score for each cell, which was then mapped onto the UMAP plot for visual representation. To further aid interpretation, the CytoTRACE tool was utilized to assess the differentiation state of the cells. Integration of cell subtype annotations, differentiation states, and sensitivity scores enabled a multidimensional correlation analysis. The results were finally summarized and displayed through composite visualizations that illustrate the relationships among cell subpopulations, differentiation status, and drug/target sensitivity^15^.

### Flow cytometry

Flow cytometry was conducted to assess cell cycle progression and apoptosis in paclitaxel-resistant TNBC cells (MDA-MB-231R and CAL-51R). Following 48h of paclitaxel treatment at indicated concentrations, cells were harvested for analysis. For cell cycle evaluation, we fixed cells overnight in 75% ethanol at 4 °C, then stained with propidium iodide (50 µg/mL, Beyotime, Cat# ST511) containing RNase A (100 µg/mL) and 0.2% Triton X-100 for 30 min at room temperature in the dark. DNA content was measured using a BD FACSVerse cytometer, and cell cycle phase distribution was determined with ModFit LT. Apoptosis was evaluated using the APC Annexin V/7-AAD kit (BioLegend, Cat# 640932). After treatment, we resuspended cells with binding buffer and mixed with APC Annexin V and 7-AAD for 15 min. Stained cells were analyzed by flow cytometry.

### Immunohistochemistry (IHC) staining and scoring

For IHC evaluation of VASN, ABCB1, IGF2BP3 and Ki67, 4 μm FFPE sections of BC specimens were processed using specific rabbit monoclonal antibodies (VASN, Abiowell AWA49995, 1:200; IGF2BP3, Abiowell AWA00962, 1:200; Ki67, AF02778, Aifang Biotechnology; ABCB1(P Glycoprotein Recombinant Rabbit, Abiowell AWA11018), anti-CEBPB (Proteintech, 83791-6-RR) along with citrate buffer antigen retrieval and detection system. We used ImageJ software (version 1.54) to quantitatively analyze IHC images. The cumulative optical density, represented as the Integrated Optical Density (IOD), was first calculated for each region of interest. Subsequently, the Average Optical Density (AOD) was derived by dividing the IOD value by the corresponding distribution area. Differences in protein expression were then estimated by comparative analysis of the AOD values.

### RNA preparation and quantitative real-time PCR (qRT-PCR)

We extracted total RNA from centrifugally precipitated cells by adding TRIzol (Invitrogen), and subsequently, the extracted RNA was reverse-transcribed into cDNA employing MCE HiScript IV reverse transcriptase (MCE004-100). Then, quantitative PCR was run on Bio-Rad CFX96 system using SYBR Premix Ex Taq™ II (MCE) and gene-specific primers (**Table S3**). We performed three biological replicates and two technical duplicates for each experimental group to ensure reproducibility. Relative gene RNA levels were normalized to GAPDH and determined using the 2^^(-ΔΔCt)^ algorithm.

### Plasmid construction and transfection

Gene knockdown vectors were constructed by inserting annealed shRNA oligonucleotides targeting VASN, ABCB1, IGF2BP3, and USP10 into the AgeI/EcoRI-digested and BsmBI-linearized pLKO.1-RFP vector. The resultant plasmids were sequence-verified (**Table S4**). Overexpression vectors were generated by PCR-amplifying the full-length coding sequences of VASN, ABCB1, and IGF2BP3 from HEK293T cDNA and cloning them into the pCDH-CMV-EF1α-CopGFP-T2A-Puro backbone (System Biosciences) via In-Fusion HD.

### Protein-protein docking analysis (HDOCK)

To investigate the potential interaction between VASN and IGF2BP3, a protein-protein docking analysis was performed using the HDOCK server. The full-length structure of VASN and the flexible region of IGF2BP3 were accurately modeled using the deep learning-based structure prediction tool AlphaFold3^16^. HDOCK is a computational tool that integrates template-based modeling and free docking algorithms for efficient prediction of intermolecular interaction modes^17^. In its refinement stage, HDOCK screens for more stable and higher-affinity interaction models by calculating the binding energy between the bait protein and the structurally aligned target.

### Western blot (WB)

Cellular proteins were harvested using RIPA lysis buffer (Beyotime, P0013B) containing protease inhibitors (Beyotime, ST506) at 4°C for 30 min. Equivalent amounts of protein lysates were fractionated via SDS-PAGE (150V, 35min) and subsequently electroblotted onto PVDF membranes (Merck Millipore, IPFL00010). Following a blocking step with 5% skim milk in TBST, the membranes were immunoblotted with specific primary antibodies at 4°C overnight. Signal detection was performed on a ChemiDoc MP system (Bio-Rad). Densitometric quantification of the blots was carried out using Image Lab 6.1 software, with the intensity of target proteins normalized to that of β-tubulin to ensure equal loading.

The following are the primary antibodies used in this study: VASN (Abiowell), CEBPB (Proteintech), ABCB1 (P-gp; Proteintech), IGF2BP3 (Proteintech), USP10 (Proteintech), p-PI3K (p85 Tyr458; Covance), PI3K (p110α; Abcam), AKT (Proteintech), p-AKT (Ser473; Proteintech), Ubiquitin (Proteintech), K48-Ub (Abcam), K63-Ub (Abcam), HA tag ( Abcam), GAPDH (Proteintech), and β-tubulin (Sigma).

### Immunofluorescence staining

For immunofluorescence analysis, cells cultured on coverslips were harvested 24 h after transfection and fixed with methanol. The preparations were then probed overnight at 4°C with a cocktail of primary antibodies, namely anti-HA (1:500; Proteintech), anti-IGF2BP3 (1:500; Abiowell), and anti-USP10 (1:100; HUABIO). After thorough rinsing, the coverslips were treated with Alexa Fluor-conjugated secondary antibodies (Life Technologies) for signal development. Nuclear staining was achieved by incubating with Hoechst 33342 (Sigma-Aldrich). Confocal micrographs were captured using a Nikon A1R system equipped with NIS-Elements software (v4.6), with all parameters kept constant across samples to guarantee reproducibility.

### Cell viability assay

To investigate the sensitivity to PTX, MDA-MB-231 and CAL-51 cell lines were inoculated into 96-well plates and cultured under standardized *in vitro* conditions. Then we treated cells with PTX at indicated concentrations for 48 hours, after which CCK-8 cell (APEx-BIO, USA) was added for a 2-hour incubation. Optical density (OD) at 450 nm was recorded using a BioTek Synergy H1 microplate reader across three separate biological replicates. The percentage of viable cells in treatment groups was calculated by comparing their absorbance readings to that of the vehicle-treated control group, with the latter being set to represent 100% viability.

### Colony formation assay

1000-1200 cells were inoculated into each well of 12-well plates and cultured for 14 days to facilitate colony development. Then, cells were fixed with 4% paraformaldehyde, followed by a staining step using Beyotime crystal violet solution (#C0121, China) under the same temperature conditions. After washing away unbound dye with distilled water, the colonies were photographed, and colony numbers were counted for subsequent quantitative analysis.

### N6-methyladenosine (m^6^A) RIP-qPCR

To identify m^6^A modifications of target genes, we performed MeRIP assays on cells using the Magna MeRIP™ m^6^A Kit (Millipore, MA, USA), following a previously established method. RNA extracted from MDA-MB-231R cells was first chemically fragmented into fragments of 100 nucleotides or less, then subjected to magnetic immunoprecipitation with an m^6^A monoclonal antibody. We used qRT-PCR to analyze the RNA fragments obtained after IP, and m^6^A enrichment was normalized to the input RNA per the manufacturer's instructions. The m^6^A modification site of ABCB1 mRNA was predicted via the RMBase v2.0 database^18^. For gene-specific m^6^A RIP-qPCR, we designed and used the following primer pairs: EEF1A positive control, EEF1A negative control, and ABCB1(shown in **Table S5**).

### RNA sequencing

We perform RNA sequencing of tissue samples of clinical TNBC breast cancer to discover key genes of paclitaxel resistance. Data supporting the findings of this paper have been submitted to the GenBase database, which is maintained by the National Genomics Data Center (NGDC) at the China National Center for Bioinformation. The accession number for this submission is CRA039413, and the data are openly available at https://ngdc.cncb.ac.cn/genbase. The clinical information of patients was presented in **Table S6**.

We conducted transcriptome sequencing on VASN-overexpressing MDA-MB-231 cells alongside vector controls. Three independent biological replicates per condition were cultured under uniform conditions (10% FBS/DMEM-F12). Total RNA was isolated with TRIzol reagent (Invitrogen), followed by DNase I treatment to eliminate genomic DNA contamination. Only samples with RNA integrity numbers (RIN) ≥8.0, as determined by an Agilent 2100 Bioanalyzer, were used for library preparation. Strand-specific sequencing libraries were constructed from 2 μg of RNA per sample using the NEBNext Ultra II kit, and subsequently sequenced on an Illumina NovaSeq 6000 platform, generating paired-end 150 bp reads with a minimum of 40 million reads per sample.

For data analysis, raw reads underwent quality trimming using fastp (threshold Phred score ≥20). The resulting clean reads were aligned to the GRCh38 human genome reference via the Rsubread package, and gene-level counts were obtained with featureCounts. Differential expression analysis between control and VASN-overexpressing groups was performed using DESeq2, with significantly differentially expressed genes (DEGs) defined by FDR < 0.05 and |log2FC| > 1. The expression patterns of these DEGs were visualized as heatmaps generated by the pheatmap package. To elucidate the biological relevance of the transcriptomic changes, we performed functional enrichment analyses, including GO and KEGG over-representation tests as well as GSEA using the MSigDB database; all enrichment analyses were carried out with the clusterProfiler package.

### Chromatin immunoprecipitation (ChIP) assay

ChIP assays were conducted on MDA-MB-231 cells using a commercial kit (Bersin Bio, China). A total of 2×10⁷ cells were collected and cross-linked with 1% formaldehyde. Nuclear extracts were prepared from the washed pellets using lysis buffer. Sheared chromatin fragments, obtained via ultrasonication, were enriched through overnight immunoprecipitation with the antibody at 4°C. After overnight reverse-crosslinking at 65°C, enriched DNA was extracted and quantified by qPCR. This assay utilized the CEBPB antibody (83791-6-RR, Proteintech), we provided ChIP-qPCR primer sequences in **Table S7** of the Supporting Information.

### Dual-luciferase reporter assay

Cells cultured in 12-well plates were co-transfected with a VASN promoter-driven firefly luciferase construct and a Renilla control plasmid. After 48 h, we used the Dual-Luciferase Reporter Assay System (Promega) to measure luciferase activities. Firefly signals were normalized to Renilla signals to account for transfection efficiency.

### Co-Immunoprecipitation (Co-IP) and Liquid Chromatography-Tandem Mass Spectrometry (LC-MS/MS)

Cellular proteins were extracted using IP lysis buffer (Beyotime, P0013) containing EDTA-free protease inhibitor cocktail (Roche, cOmplete). Following clarification via centrifugation, the supernatants were incubated overnight at 4°C with rotation in the presence of anti-HA magnetic beads (MedChemExpress, HY-K0201) or isotype control IgG (Beyotime, A7016). After removal of unbound materials, bound proteins were eluted and divided for downstream applications: one aliquot was resolved by SDS-PAGE on gradient gels (Bio-Rad, #4561094) followed by ECL Prime detection (Biosharp), while the other was submitted to Cosmos Wisdom (Hangzhou, China) for LC-MS/MS analysis.

### Xenograft studies

All animal procedures were approved by the Institutional Animal Care and Use Committee of Chongqing Medical University (Approval ID: IACUC-CQMU-2025-11078) and conducted in compliance with ARRIVE guidelines. All animal experiments were conducted using four-week-old female BALB/c nude mice housed under SPF conditions.

For proliferation assays, MDA-MB-231-shCtrl or -shVASN cells (5 × 10⁶) suspended in 1:1 PBS/Matrigel (BD Biosciences) were orthotopically implanted into the fourth mammary fat pads. Tumor growth was monitored for 42 days.

For drug response evaluation, mice bearing MDA-MB-231 xenografts (~400 mm^3^) were randomized to receive either vehicle or paclitaxel (5 mg/kg, i.p., once every two days for five cycles; MCE #HY-B0015) (n = 5 per group).

In subsequent combination therapy studies, mice implanted with MDA-MB-231R or CAL-51R cells (5 × 10⁶) were randomized upon reaching ~400 mm³ using the Microsoft Excel RAND algorithm into four groups (n = 5 each): vehicle control, paclitaxel monotherapy (5 mg/kg every two days, i.p., five cycles), Trametinib monotherapy (1 mg/kg/day, oral gavage; MCE #HY-10999), or combination therapy (Trametinib for 14 consecutive days with concurrent paclitaxel administration for five cycles). Outcome assessments were performed by investigators blinded to the group assignments.

To quantitatively evaluate the synergistic interaction between paclitaxel and Trametinib *in vivo*, we applied the Bliss independence model. First, the tumor growth inhibition rate (fraction affected, Fa) for each treatment group was calculated using the following formula: Fa = 1 - (T/C), where T (test) and C (control) are mean tumor volumes at the endpoint. Under the Bliss independence model, expected additive inhibition (E_add) under Bliss independence: E_add= FaA+FaB-FaA*FaB, where FaA and FaB are the inhibition rates of monotherapy A (paclitaxel) and monotherapy B (Trametinib), respectively. The synergy score was then defined as: Synergy score= Facomb/E_add. A synergy score> 1 indicates that the two drugs exhibit a synergistic effect at this concentration. A synergy score= 1 indicates that the two drugs exert only an additive effect. A synergy score< 1 indicates that the two drugs show an antagonistic interaction.

### Drug screening

This study screened VASN-associated therapeutic drugs using the oncoPredict R package (v1.2.0) and CTRPv2 dataset (retrieved from https://osf.io/742q5/), including gene expression and drug response matrices. All analyses were performed in R 4.4.0, with dplyr, tidyr, and ggplot2 for data processing and visualization. Data preprocessing included matching common cell lines, constructing a VASN expression marker matrix, and imputing missing values with medians. The GLDS algorithm in oncoPredict was used to analyze VASN-drug associations. Significant drugs were screened by p-value < 0.05 and sorted by p-value and statistic score.

### Molecular Dynamics (MD) simulations

The system investigated in this study is a protein-ligand complex comprising a protein receptor and a small-molecule ligand. All simulations were performed using the GROMACS software package, following the procedures outlined below. The protein structure file was imported into GROMACS. The topology file and the simulation box were generated using the pdb2gmx and gmx editconf utilities. The structure was subjected to energy minimization using the gmx grompp and gmx mdrun modules. A 100 ns all-atom molecular dynamics simulation was carried out with gmx grompp and gmx mdrun, during which conformational snapshots were saved at regular intervals for subsequent analysis. The root-mean-square deviation (RMSD), root-mean-square fluctuation (RMSF), radius of gyration, solvent-accessible surface area (SASA), and number of hydrogen bonds of the protein were computed using the gmx rms and related GROMACS analysis tools. The results were visualized through plots and summarized in statistical tables.

### Cellular Thermal Shift Assay (CETSA)

Cellular Thermal Shift Assay was performed to assess the binding of Trametinib to VASN in cells. Briefly, cells treated with vehicle (DMSO) or Trametinib were aliquoted and heated at a temperature gradient (28-77°C for preliminary experiments, and 37-57°C for formal validation) for 3 min. Cells were then lysed, and the soluble fractions were collected after centrifugation. The remaining soluble VASN protein levels were detected by Western blotting, with β-Tubulin used as a loading control.

### Statistical analysis

All statistical analyses were performed using GraphPad Prism 9 software (Research Resource Identifier: SCR_002798). The analytical approaches were tailored to the experimental design: one-way analysis of variance (ANOVA) was utilized for comparisons involving three or more independent groups, whereas unpaired two-tailed Student's t-tests were applied for pairwise comparisons between two distinct groups. For matched sample analyses (e.g., paired cancerous and adjacent non-tumorous breast tissues), paired Student's t-tests were implemented to account for intragroup correlations. Statistical significance was universally defined as a p-value < 0.05 for all comparisons. To guarantee the reliability and reproducibility of experimental data, all assays were conducted with at least three independent biological replicates (n ≥ 3).

## Results

### VASN was identified as a key candidate for paclitaxel resistance in TNBC

**Figure 1:**
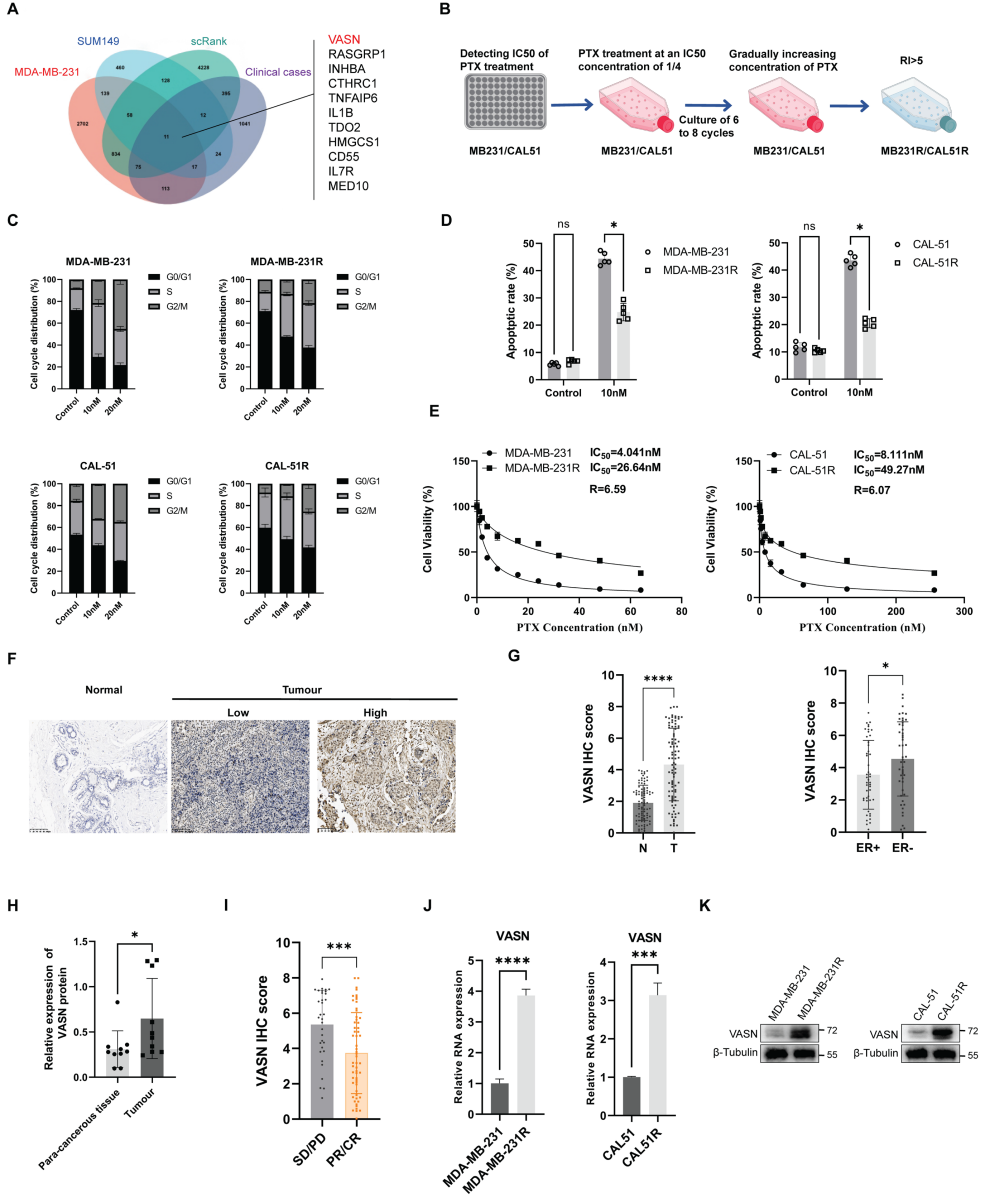
** VASN is upregulated in paclitaxel-resistant TNBC. (A)** Venn diagram showing the overlap of differentially expressed genes (DEGs) from three datasets: TNBC patients with neoadjuvant chemotherapy resistance, paclitaxel-resistant TNBC cells (GSE90564) (|log_2_FC| > 1, adjusted P< 0.05), and scRank-based drug sensitivity analysis (GSE169246). VASN is highlighted as a key overlapping candidate. **(B)** Schematic representation of the stepwise establishment of paclitaxel-resistant TNBC sublines (MDA-MB-231R and CAL-51R) from parental MDA-MB-231 and CAL-51 cells via exposure to increasing concentrations of paclitaxel. **(C)** Flow cytometry analysis of cell cycle distribution in parental and paclitaxel-resistant TNBC cells treated with paclitaxel for 24 h, n = 5 in each group. **(D)** Flow cytometry analysis of apoptosis in parental and paclitaxel-resistant TNBC cells treated with paclitaxel for 48 h. ns, not significant; *P < 0.05, n = 5 in each group. **(E)** Cell viability assays showing paclitaxel sensitivity in parental and resistant TNBC sublines. **(F)** Representative immunohistochemistry (IHC) images of VASN expression in breast cancer tissues. **(G)** Quantification of VASN IHC scores in normal (N) vs. tumor (T) breast tissues (left) and in ER-positive vs. ER-negative breast cancer subtypes (right). *P < 0.05, ****P < 0.0001. **(H)** Western blot analysis of VASN protein expression in adjacent normal tissues and tumor tissues from TNBC patients, with quantification showing significant upregulation in tumors. *P < 0.05. **(I)** VASN IHC scores in TNBC patients stratified by response to paclitaxel-based therapy. ***P < 0.001. **(J)** Relative VASN mRNA expression in parental and paclitaxel-resistant TNBC sublines, as determined by qRT-PCR, n = 5 in each group. ****P < 0.0001, ***P < 0.001, with GAPDH as a control. **(K)** Western blot analysis of VASN protein expression in parental and paclitaxel-resistant TNBC sublines, with GAPDH as a loading control.

To screen key genes driving paclitaxel resistance in TNBC, we obtained the overlapping differentially expressed genes (DEGs) from TNBC patients with resistance to neoadjuvant chemotherapy, paclitaxel-resistant TNBC cells (GSE90564) and scRank-based drug sensitivity analysis (GSE169246). Intersection analysis revealed VASN as one of the prominent overlapping candidates (**Fig. 1A; Fig. S1A**). To functionally validate the role of VASN in paclitaxel resistance, we established stable paclitaxel-resistant sublines (MDA-MB-231R and CAL-51R) from parental TNBC cells via stepwise exposure to increasing paclitaxel concentrations (**Fig. 1B**). Flow cytometry demonstrated that paclitaxel-induced apoptosis and G₂/M phase cell cycle arrest were significantly attenuated in resistant cells (**Fig. 1C, D; Fig. S1B, C**). Cell viability assays further confirmed a 6.59-fold increase in paclitaxel IC₅₀ in MDA-MB-231R (from 4.64 nM to 26.44 nM) and a 6.07-fold increase in CAL-51R (from 8.11 nM to 49.27 nM), thus confirming the successful establishment of paclitaxel-resistant cell models (**Fig. 1E**).

Consistently, clinical validation using cancer patient specimens confirmed that VASN protein was significantly higher in tumor tissues than in adjacent normal tissues, and was further elevated in ER-negative subtypes compared to ER-positive tumors (**Fig. 1F, G**). This upregulation was validated at the protein level using protein extracted from fresh clinical TNBC patient specimens (**Fig. 1H; Fig. S1D**). Importantly, patients with stable/progressive disease (SD/PD) following paclitaxel-based therapy exhibited significantly higher VASN IHC scores and protein levels than those with partial/complete response (PR/CR) or pathological complete response (pCR) (**Fig. 1I; Fig. S1E, F**). VASN mRNA and protein levels were significantly elevated in both MDA-MB-231R and CAL-51R resistant sublines compared to their parental counterparts (**Fig. 1J, K**).

Together, these results identified VASN as a potential driver of paclitaxel resistance in TNBC, linking its upregulation in resistant cells and clinical specimens to poor therapeutic response.

### VASN enhances the proliferative capacity of TNBC cells and confers paclitaxel resistance

**Figure 2:**
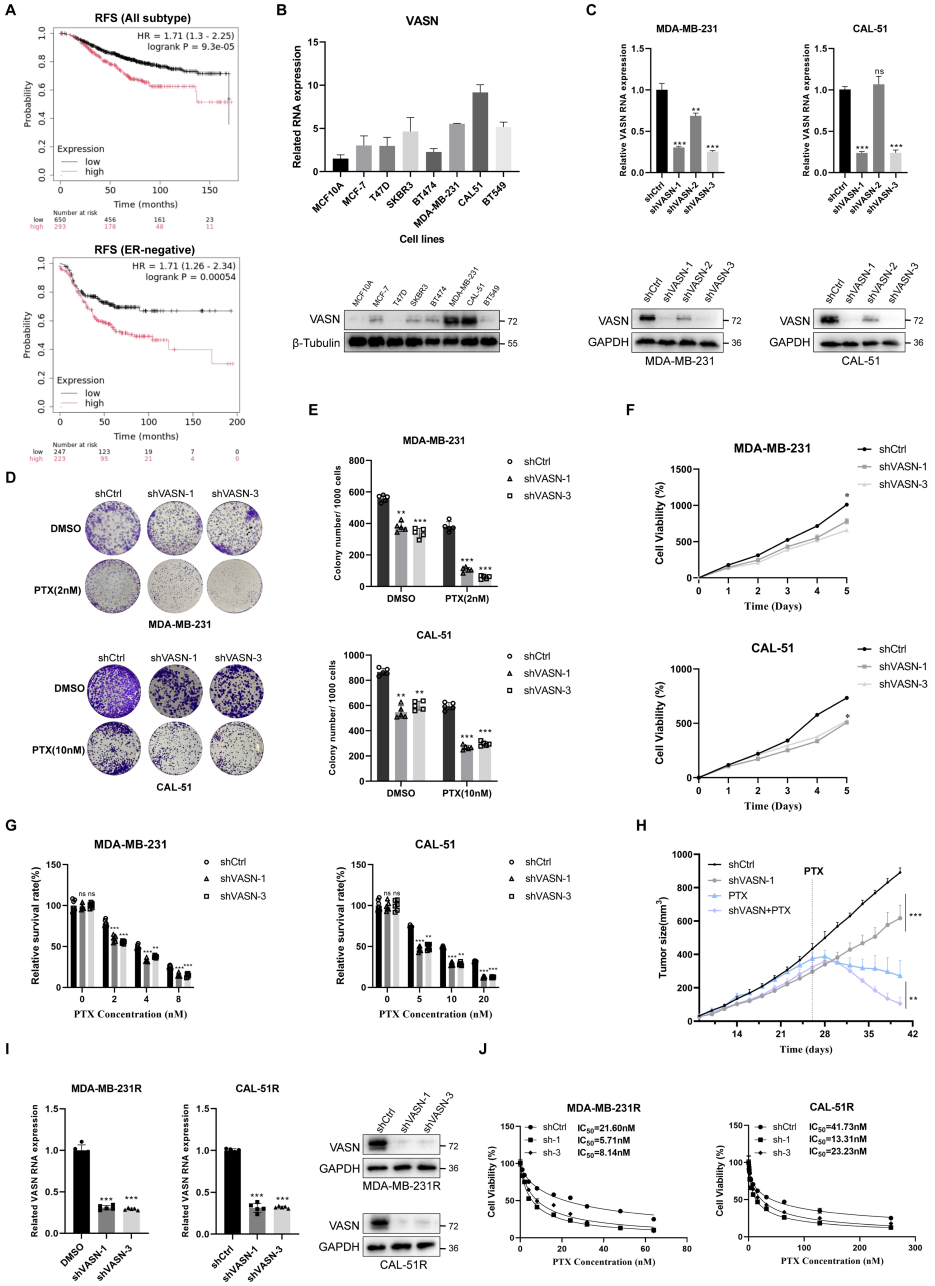
** VASN promotes TNBC cell proliferation and confers paclitaxel resistance. (A)** Relapse-free survival (RFS) curves for breast cancer patients stratified by VASN expression (low vs. high) in all subtypes (top) and ER-negative subtypes (bottom). HR, hazard ratio; log-rank P-values are indicated. **(B)** VASN mRNA (top) and protein (bottom) expression in a panel of breast cancer cell lines. β-tubulin was used as a loading control, n = 3 in each group. **(C)** Validation of VASN knockdown efficiency in MDA-MB-231 and CAL-51 cells transduced with three independent shRNAs (shVASN-1, shVASN-2, shVASN-3) at both mRNA (top) and protein (bottom) levels, relative to shCtrl. GAPDH was used as a loading control, n = 3 in each group. **(D)** Representative colony formation images of shCtrl and VASN-knockdown MDA-MB-231 and CAL-51 cells treated with DMSO or paclitaxel (PTX). **(E)** Quantification of colony formation in shCtrl and VASN-knockdown TNBC cells treated with DMSO or paclitaxel. Data are presented as mean ± SD, n = 5 in each group. **P < 0.01, ***P < 0.001, ****P < 0.0001. **(F)** Cell proliferation curves of shCtrl and VASN-knockdown MDA-MB-231 and CAL-51 cells over 5 days, showing reduced growth in knockdown cells, n = 5 in each group. **(G)** Relative survival rates of shCtrl and VASN-knockdown MDA-MB-231 and CAL-51 cells treated with increasing concentrations of paclitaxel, n = 5 in each group. **(H)** *In vivo* tumor growth curves of MDA-MB-231 xenografts in nude mice treated with shCtrl, shVASN-1, paclitaxel, or shVASN-1 + PTX. Tumor volumes were measured at the indicated time points, n = 5 in each group. Data are presented as mean ± SD. **(I)** Validation of VASN knockdown in paclitaxel-resistant MDA-MB-231R and CAL-51R cells at mRNA (top) and protein (bottom) levels, relative to shCtrl. GAPDH was used as a loading control, n = 5 in each group. **(J)** Cell viability assays of shCtrl and VASN-knockdown MDA-MB-231R and CAL-51R cells treated with increasing concentrations of paclitaxel, showing partial restoration of chemosensitivity, n = 4 in each group. IC₅₀ values are indicated.

To evaluate the prognostic significance of VASN in breast cancer patients, we conducted survival analysis based on the kmplot database (https://kmplot.com/analysis/). The results revealed that high VASN expression was significantly correlated with inferior relapse-free survival (RFS) in breast cancer patients, especially in the ER-negative subtype (**Fig. 2A**). We detected VASN expression levels across a panel of breast cancer cell lines and found that both mRNA and protein levels of VASN were markedly upregulated in TNBC cell lines (**Fig. 2B**).

Subsequently, we established stable VASN-knockdown MDA-MB-231 and CAL-51 cell lines (**Fig. 2C**). VASN knockdown remarkably inhibited colony formation and cell proliferation, while sensitizing TNBC cells to paclitaxel (**Fig. 2D-G**). *In vivo* xenograft models further confirmed that VASN knockdown combined with paclitaxel treatment resulted in the slowest tumor growth (**Fig. 2H; Fig. S2A**).

In contrast, VASN overexpression enhanced cell proliferation and reduced paclitaxel sensitivity (**Fig. S2B-F**). Paclitaxel treatment markedly increased intracellular ROS levels and induced oxidative damage to critical cellular components. Notably, the elevation of ROS was substantially blunted in VASN-overexpressing TNBC cells upon paclitaxel exposure (**Fig. S2G**). Furthermore, VASN knockdown in paclitaxel-resistant MDA-MB-231R and CAL-51R cells partially restored cellular sensitivity to paclitaxel (**Fig. 2I, J**). Collectively, these findings demonstrate that VASN acts as a critical regulator of TNBC progression and chemoresistance. VASN knockdown not only suppresses tumor growth but also re-sensitizes paclitaxel-resistant TNBC cells to chemotherapy.

### CEBPB upregulates VASN expression by directly binding to its promoter

**Figure 3:**
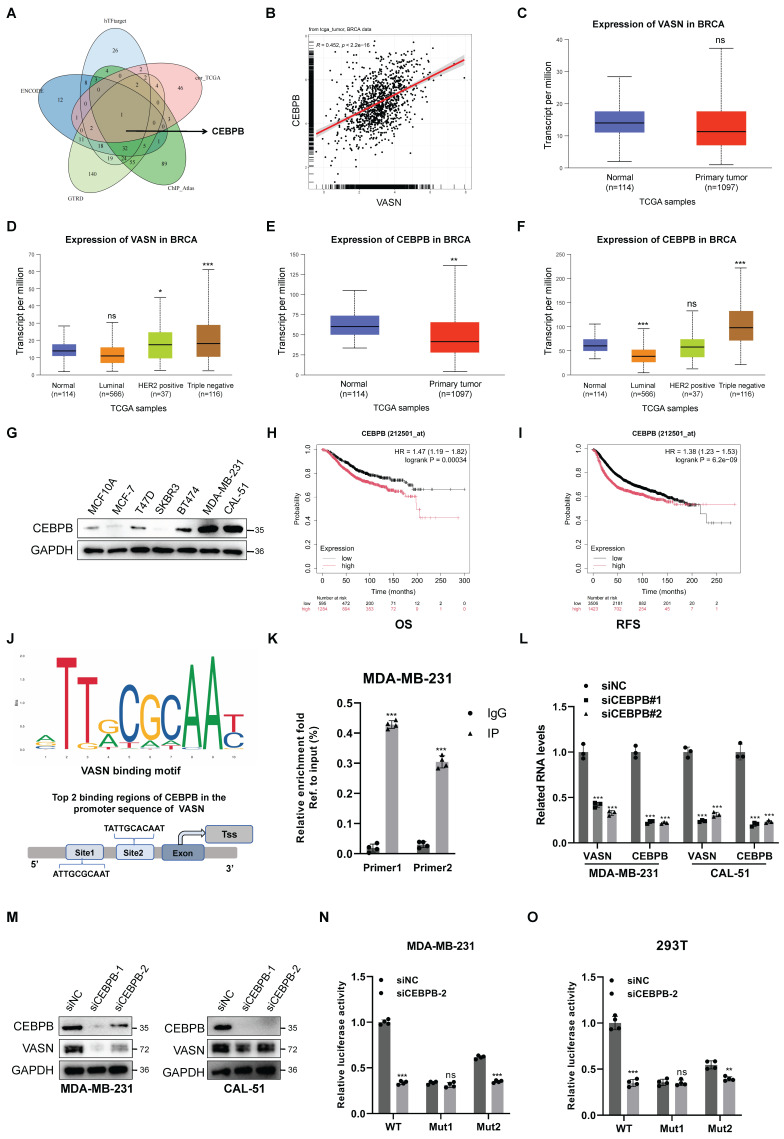
** CEBPB upregulates VASN expression by directly binding to its promoter. (A)** Venn diagram showing the overlap of transcription factors (TFs) predicted to regulate VASN from four databases (hTFtarget, GTRD, ENCODE, ChIP-ATLAS), identifying CEBPB as a key candidate. **(B)** Scatter plot showing a positive correlation between CEBPB and VASN mRNA expression in the TCGA breast cancer cohort (n = 1097). **(C)** Box plot of VASN mRNA expression in normal (n=114) vs. primary tumor (n=1097) breast tissues from TCGA. ****P < 0.0001. **(D)** Box plot of VASN mRNA expression in normal breast tissues and breast cancer molecular subtypes (Luminal A, Luminal B, HER2-positive, triple-negative) from TCGA. ****P < 0.0001. **(E)** Box plot of CEBPB mRNA expression in normal (n=114) vs. primary tumor (n=1097) breast tissues from TCGA. ****P < 0.0001. **(F)** Box plot of CEBPB mRNA expression in normal breast tissues and breast cancer molecular subtypes from TCGA. ****P < 0.0001. **(G)** Western blot analysis of CEBPB protein expression in a panel of breast cell lines: non-tumorigenic MCF-10A, ER-positive (MCF-7, T47D, BT474), and TNBC (MDA-MB-231, CAL-51) cells. GAPDH was used as a loading control. **(H, I)** Kaplan-Meier curves showing overall survival (OS) and relapse-free survival (RFS) of breast cancer patients stratified by CEBPB expression (low vs. high). HR, hazard ratio; log-rank P-values are indicated. **(J)** Schematic representation of the VASN promoter region, highlighting the conserved CEBPB-binding motif (ATTGCGCAAT) and two binding sites (Site 1 and Site 2) used for ChIP-qPCR analysis. **(K)** ChIP-qPCR analysis confirming direct binding of CEBPB to the VASN promoter in MDA-MB-231 cells. Data are presented as fold enrichment relative to IgG control, n = 4 in each group. **(L)** qRT-PCR analysis of VASN and CEBPB mRNA expression in MDA-MB-231 and CAL-51 cells transfected with control siRNA (siNC) or two independent CEBPB siRNAs (siCEBPB#1, siCEBPB#2), n = 3 in each group. **(M)** Western blot analysis of CEBPB and VASN protein expression in MDA-MB-231 and CAL-51 cells transfected with siNC, siCEBPB#1, or siCEBPB#2. GAPDH was used as a loading control. **(N, O)** Dual-luciferase reporter assays in MDA-MB-231 and 293T cells showing the effect of mutating the core CEBPB-binding site (Mut1) on VASN promoter activity, compared to wild-type (WT) and a control mutation (Mut2), n = 4 in each group. ns, not significant; *P < 0.05.

To elucidate the mechanism underlying elevated VASN expression in breast cancer, particularly in TNBC, we sought to identify upstream transcription factors regulating VASN transcription. Differential transcription factor expression in breast cancer was first analyzed using TCGA database. We then integrated these findings with data from hTFtarget, GTRD, ENCODE, and ChIP-ATLAS databases to screen for transcription factors potentially activating VASN expression. This multi-database approach identified CEBPB as the predominant overlapping transcription factor (**Fig. 3A**). Correlation analysis revealed a strong positive association between CEBPB and VASN expression in the TCGA breast cancer cohort, suggesting a potential regulatory role for CEBPB in VASN transcription (**Fig. 3B**). Consistent with VASN expression patterns, CEBPB was markedly upregulated in breast cancer tissues compared to normal counterparts, with the highest expression observed in TNBC subtypes (**Fig. 3C-F**). IHC staining further confirmed elevated CEBPB protein levels in breast tumor tissues relative to adjacent normal tissues (**Fig. S3A**), with particularly pronounced expression in estrogen receptor (ER)-negative tumors(**Fig. S3B-C**). Consistently, CEBPB protein expression was elevated in TNBC cell lines (MDA-MB-231, CAL-51) compared to non-tumorigenic MCF-10A and ER-positive breast cancer cells (MCF-7, T47D, BT474) (**Fig. 3G; Fig. S3D**). Survival analysis demonstrated that high CEBPB expression was associated with poorer overall survival (OS) and RFS in breast cancer patients (**Fig. 3H-I**).

Subsequently, we used the JASPAR database to predict CEBPB binding motifs in the VASN promoter region, identifying a conserved CEBPB-binding motif (ATTGCGCAAT) in the proximal promoter of VASN (**Fig. 3J**). ChIP assays confirmed that CEBPB could directly bind to the predicted site in the VASN promoter in MDA-MB-231 cells (**Fig. 3K**). CEBPB knockdown via siRNA significantly reduced VASN mRNA and protein levels in MDA-MB-231 and CAL-51 cells (**Fig. 3L-M**). Dual-luciferase reporter assays further validated that mutation of the core CEBPB-binding site (Mut1) significantly attenuated the transcriptional activity of the VASN promoter, while Mut2 had no obvious effect (**Fig. 3M-N**), indicating that this site is critical for CEBPB-mediated VASN transcription.

In functional assays, we found that CEBPB knockdown significantly increased the sensitivity of MDA-MB-231 and CAL-51 cells to paclitaxel (**Fig. S3E**). Furthermore, in paclitaxel-resistant TNBC cell lines (MDA-MB-231R and CAL-51R), CEBPB knockdown rescued sensitivity to paclitaxel, as reflected by reduced cell viability and impaired colony-forming ability (**Fig. S3F-I**). Taken together, these findings confirm that CEBPB directly binds to the VASN promoter to upregulate its expression, thereby driving TNBC progression and mediating paclitaxel resistance.

### VASN promotes paclitaxel resistance activates the PI3K-AKT signaling pathway in TNBC

**Figure 4:**
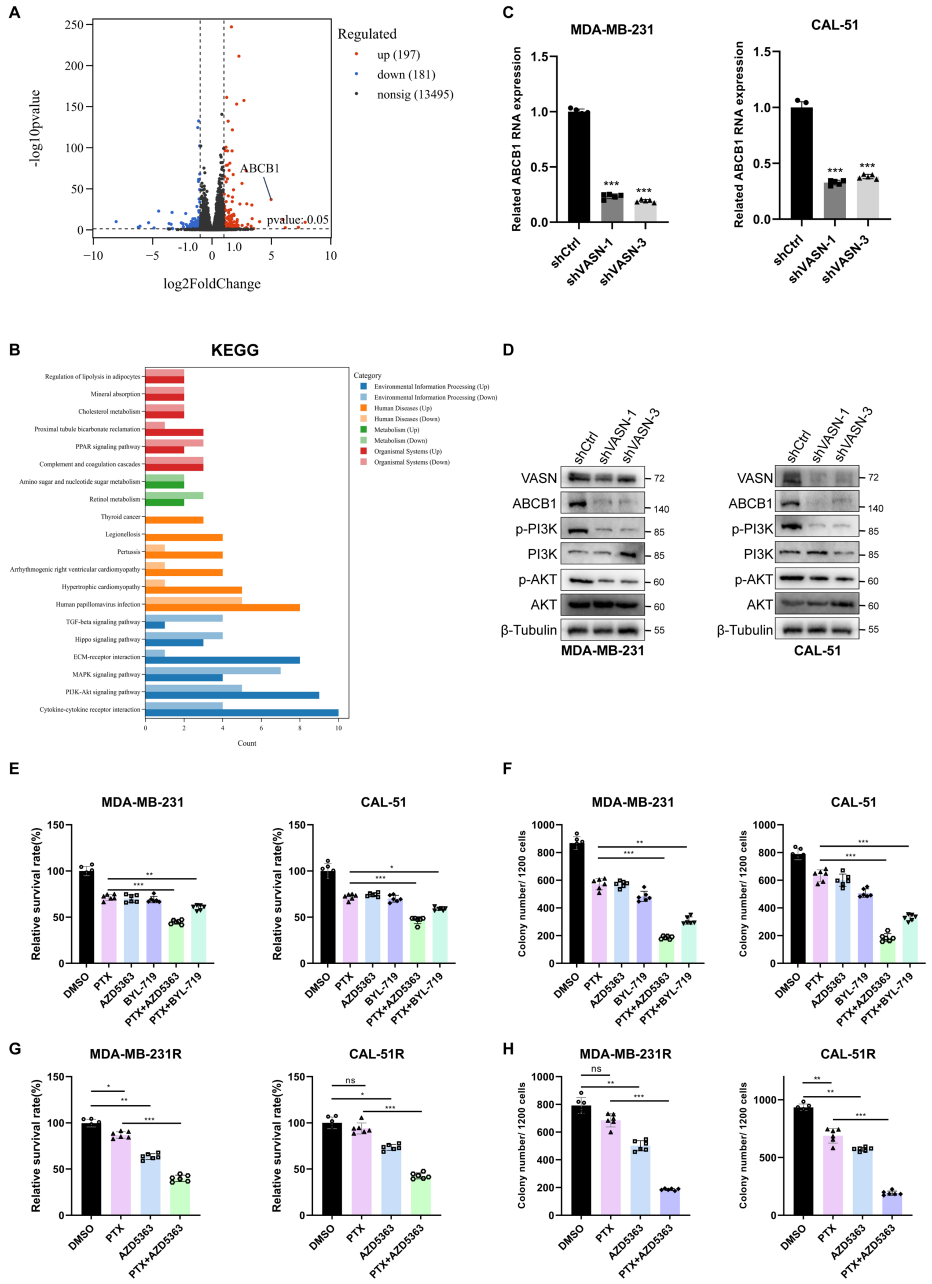
** Overexpression of VASN activates the PI3K-AKT pathway and upregulates ABCB1 in TNBC. (A)** Volcano plot of differentially expressed genes (DEGs) from RNA sequencing of VASN-overexpressing MDA-MB-231 cells compared to controls. Red dots represent upregulated genes, blue dots represent downregulated genes, and black dots indicate non-significant changes. **(B)** KEGG pathway enrichment analysis of upregulated DEGs, showing significant enrichment in the PI3K-AKT signaling pathway (among other pathways), which is highlighted as a central axis mediating VASN function. **(C)** qRT-PCR analysis of ABCB1 mRNA expression in MDA-MB-231 and CAL-51 cells transfected with shCtrl, shVASN-1, or shVASN-3. Data are normalized to shCtrl and presented as mean ± SD, n = 5 in each group. ***P < 0.001. **(D)** Western blot analysis of VASN, ABCB1, phosphorylated PI3K (p-PI3K), total PI3K (PI3K), phosphorylated AKT (p-AKT), and total AKT (AKT) protein levels in VASN-knockdown MDA-MB-231 and CAL-51 cells. β-Tubulin serves as a loading control. **(E)** Relative cell survival rates of parental MDA-MB-231 and CAL-51 cells treated with DMSO, paclitaxel (PTX) alone, PTX combined with PI3K inhibitor (BYL-719), or PTX combined with AKT inhibitor (AZD5363), n = 5 in each group. **P < 0.01, ***P < 0.001. **(F)** Quantification of colony formation in parental MDA-MB-231 and CAL-51 cells under the same treatment conditions as in (E), n = 5 in each group. ***P < 0.001. **(G)** Relative cell survival rates of paclitaxel-resistant MDA-MB-231R and CAL-51R cells treated with DMSO, PTX alone, or PTX combined with AZD5363, n = 5 in each group. **P < 0.01, ***P < 0.001. **(H)** Quantification of colony formation in paclitaxel-resistant MDA-MB-231R and CAL-51R cells under the same treatment conditions as in (G), n = 5 in each group. **P < 0.01, ***P < 0.001.

To further delineate the molecular mechanisms underlying VASN-mediated paclitaxel resistance in TNBC, we performed RNA sequencing on VASN-overexpressing MDA-MB-231 cells. ABCB1, a well-characterized paclitaxel resistance-related gene, exhibited the strongest positive correlation with VASN among the differentially expressed genes (DEGs) (filtered by both adjusted P-value and fold change) (**Fig. 4A**). Beyond this, Gene Ontology (GO) enrichment analysis revealed that upregulated genes were enriched in biological processes (BP) including angiogenesis, cell-cell adhesion, and cardiovascular pathologies. At the cellular component (CC) level, these genes mapped to pathways governing extracellular matrix (ECM) composition and remodeling. Correspondingly, molecular function (MF) enrichment terms corresponded to ECM components and adhesion molecules. Consistently, KEGG pathway analysis identified significant enrichment in the PI3K/AKT signaling pathway, providing a central signaling axis that may integrate these ECM- and adhesion-related processes to drive VASN-mediated paclitaxel resistance (**Fig. 4B, Fig. S4A**).

Consistently qRT-PCR and WB showed that VASN knockdown reduced ABCB1 mRNA and protein expression levels in MDA-MB-231/CAL-51 cells, while VASN overexpression elevated ABCB1 mRNA and protein levels (**Fig. 4C; Fig. S4B**). Besides, VASN expression levels are positively correlated with the phosphorylation of PI3K and AKT (p-PI3K/p-AKT) (**Fig. 4D; Fig. S4C**). To verify the functional relevance of this axis, we treated cells with PI3K inhibitor (BYL-719) or AKT inhibitor (AZD5363). Previous studies have demonstrated that the PI3K/AKT pathway mediates the transcription of ABCB1^19^. We therefore examined alterations in ABCB1 mRNA and protein levels following inhibition of the PI3K/AKT pathway in TNBC cell lines. Our results revealed that inhibition of the PI3K/AKT pathway led to inconsistent changes in ABCB1 expression, which were far less pronounced than the reduction observed upon VASN knockdown (**Fig. S4D, E**). Subsequently, to investigate whether inhibiting this PI3K/AKT pathway could rescue sensitivity to paclitaxel, we conducted cell proliferation and colony formation assays in the parental MDA-MB-231 and CAL-51 cells. The results showed that the combination of PI3K/AKT pathway inhibitors with paclitaxel effectively inhibited cell proliferation and colony formation, with AZD5363 demonstrating a superior effect (**Fig. 4E-F; Fig. S4F**). Furthermore, in both resistant cell lines, combined treatment with AZD5363 and paclitaxel also exhibited strong inhibitory effects (**Fig. 4G-H; Fig. S4G**).

Collectively, these results indicate that VASN activates the PI3K-AKT signaling pathway, and that inhibiting this pathway effectively restores sensitivity to paclitaxel.

### VASN drives ABCB1 transcription to mediate paclitaxel resistance in TNBC

**Figure 5:**
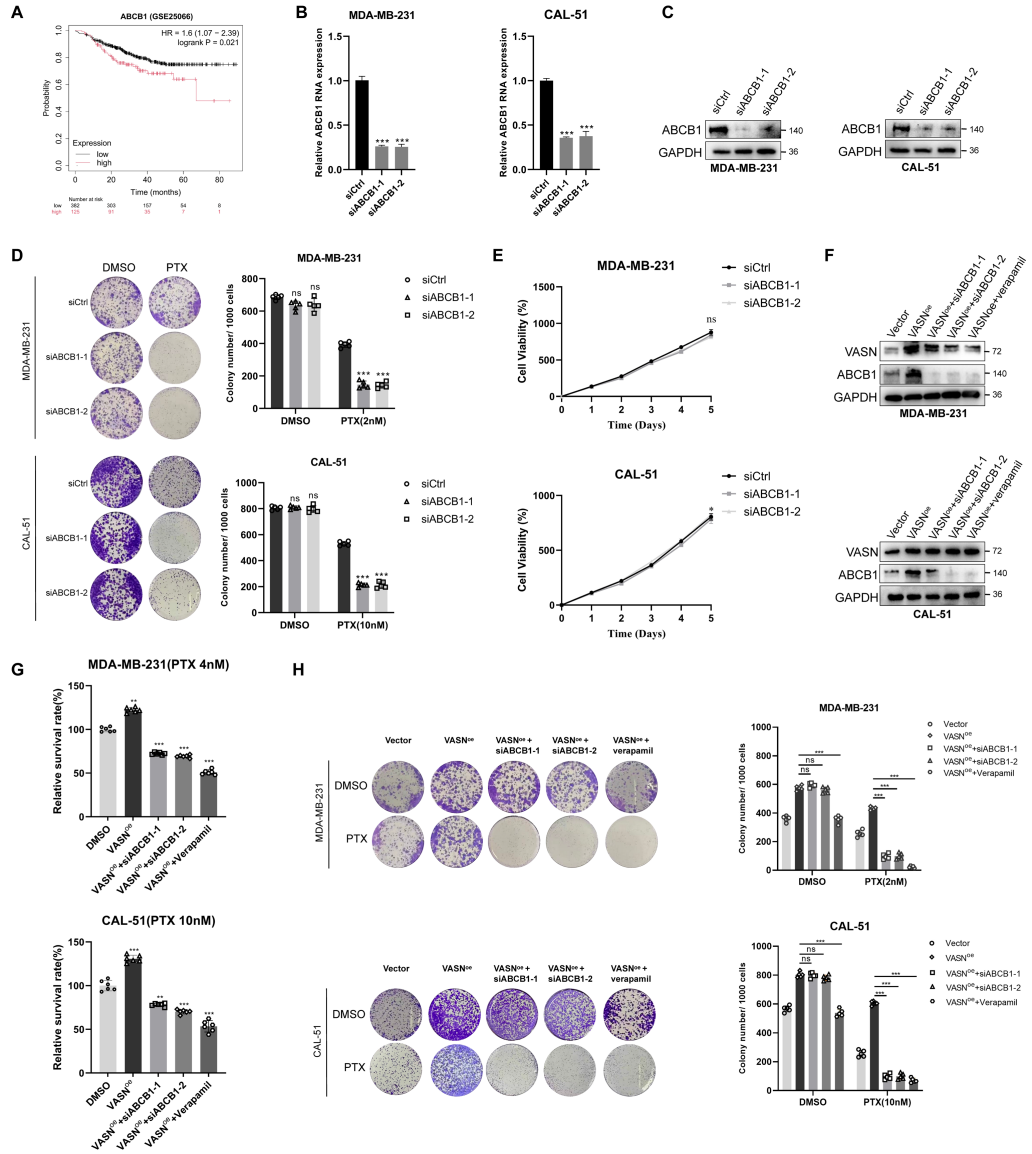
** ABCB1 is a critical downstream effector of VASN-mediated paclitaxel resistance in TNBC. (A)** Kaplan-Meier overall survival (OS) curves for TNBC patients stratified by ABCB1 expression (low vs. high). HR, hazard ratio; log-rank P-value is indicated. **(B)** qRT-PCR analysis of ABCB1 mRNA expression in MDA-MB-231 and CAL-51 cells transfected with control siRNA (siCtrl) or two independent ABCB1 siRNAs (siABCB1-1, siABCB1-2). Data are normalized to siCtrl and presented as mean ± SD, n = 5 in each group. **P < 0.01, ***P < 0.001. **(C)** Western blot validation of ABCB1 knockdown efficiency in MDA-MB-231 and CAL-51 cells transfected with siCtrl, siABCB1-1, or siABCB1-2. GAPDH was used as a loading control. **(D)** Representative colony formation images (top) and quantification (bottom) of MDA-MB-231 and CAL-51 cells transfected with siCtrl, siABCB1-1, or siABCB1-2 and treated with DMSO or paclitaxel (PTX). Silencing ABCB1 significantly enhanced paclitaxel sensitivity, n = 5 in each group. ***P < 0.001. **(E)** Cell proliferation curves of MDA-MB-231 and CAL-51 cells transfected with siCtrl, siABCB1-1, or siABCB1-2, showing no significant effect on proliferation. ns, not significant, n = 5 in each group. **(F)** Western blot analysis of VASN and ABCB1 protein expression in VASN-overexpressing MDA-MB-231 and CAL-51 cells transfected with siCtrl, siABCB1-1, siABCB1-2, or treated with verapamil (ABCB1 inhibitor). GAPDH was used as a loading control. **(G)** Relative survival rates of VASN-overexpressing MDA-MB-231 and CAL-51 cells under the indicated treatments, n = 6 in each group. **P < 0.01, ***P < 0.001. **(H)** Representative colony formation images (left) and quantification (right) of VASN overexpressing MDA-MB-231 and CAL-51 cells in response to genetic knockdown or pharmacological inhibition of ABCB1, n = 5 in each group. ***P < 0.001.

To verify that ABCB1 functions as a key downstream effector in VASN-mediated paclitaxel resistance, we first analyzed its clinical relevance using the kmplot database. The results revealed that TNBC patients with high ABCB1 expression had a significantly shorter overall survival than those with low expression (**Fig. 5A**). Subsequently, in both MDA-MB-231 and CAL-51 cell lines, we established ABCB1-knockdown models using siRNA and generated stable ABCB1-overexpressing cell lines via lentiviral transduction (**Fig. 5B-C; Fig. S5A**). Functional assays showed that, in MDA-MB-231 and CAL-51 cells, silencing ABCB1 did not markedly affect colony-forming or proliferative capacity compared to the control group. However, it significantly increased cellular sensitivity to paclitaxel (**Fig. 5D-E**). Consistently, overexpression of ABCB1 significantly enhanced paclitaxel resistance without affecting cellular proliferation (**Fig. S5B-C**). To directly confirm ABCB1 as the downstream mediator of VASN, we either silenced ABCB1 or used the ABCB1 inhibitor (verapamil) in VASN-overexpressing cell lines (MDA-MB-231 and CAL-51).

Both approaches partially rescued the increase in paclitaxel resistance conferred by VASN overexpression **(Fig. 5F-H)**. Similarly, overexpressing ABCB1 in VASN-knockdown cells (MDA-MB-231 and CAL-51) partially rescued the increased paclitaxel sensitivity resulting from VASN knockdown (**Fig. S5D-F**). Taken together, these results further confirm that ABCB1 is a critical downstream effector molecule in VASN-mediated paclitaxel resistance in TNBC.

### VASN-IGF2BP3 interaction modulates ABCB1 mRNA stability through m^6^A methylation in TNBC

**Figure 6:**
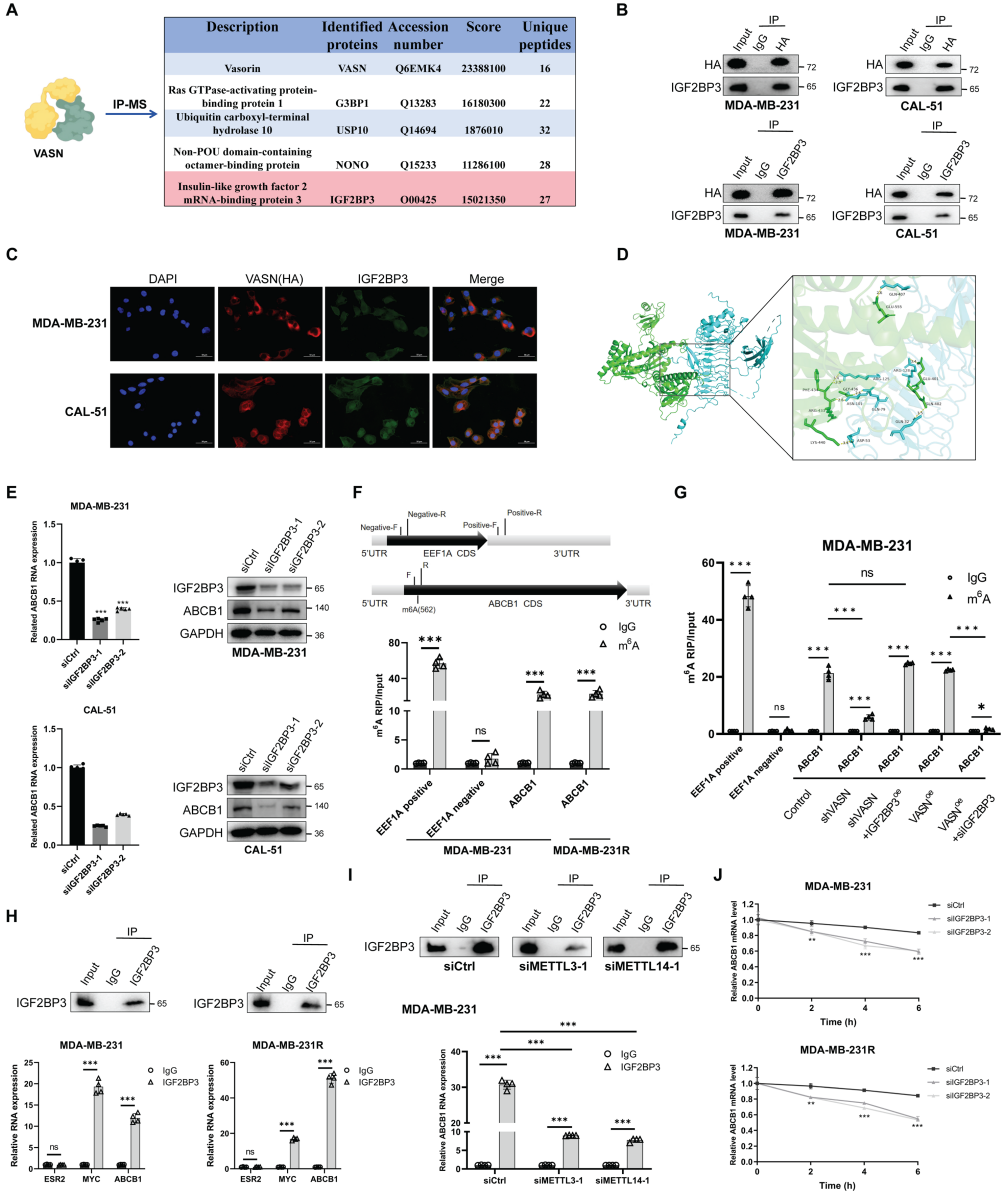
** VASN interacts with IGF2BP3 to enhance ABCB1 mRNA stability via m^6^A methylation in TNBC.** Co-immunoprecipitation followed by mass spectrometry (IP-MS) identified VASN-interacting proteins in VASN-overexpressed MDA-MB-231 cells, with IGF2BP3 as a high-scoring candidate. **(B)** Co-immunoprecipitation (Co-IP) assays confirmed the direct physical interaction between VASN and IGF2BP3 in MDA-MB-231 and CAL-51 cells. **(C)** Immunofluorescence staining showed the colocalization of VASN (HA-tagged, red) and IGF2BP3 (green) in the cytoplasm of MDA-MB-231 and CAL-51 cells. DAPI (blue) was used for nuclear staining. **(D)** Computational prediction of the 3D binding interface between VASN and IGF2BP3, highlighting key interaction residues. **(E)** (Left) qRT-PCR analysis of ABCB1 mRNA expression in MDA-MB-231 and CAL-51 cells transfected with control siRNA (siCtrl) or two independent IGF2BP3 siRNAs (siIGF2BP3-1, siIGF2BP3-2). ***P < 0.001. (Right) Western blot analysis of IGF2BP3 and ABCB1 protein expression in MDA-MB-231 and CAL-51 cells transfected with siCtrl, siIGF2BP3-1, or siIGF2BP3-2. GAPDH was used as a loading control, n = 5 in each group.**(F)** m^6^A RNA immunoprecipitation (RIP)-qPCR confirmed significant m^6^A modification in the coding sequence (CDS) region of ABCB1 mRNA in both MDA-MB-231 and MDA-MB-231R cells. EEF1A1 was used as a control (stop codon region as positive control, exon 5 as negative control), n = 4 in each group. ***P < 0.001. **(G)**Validation of m⁶A modification on ABCB1 mRNA regulated by VASN and IGF2BP3 in MDA-MB-231 cells, n = 4 in each group. ***P < 0.001. **(H)** RIP-qPCR demonstrated that IGF2BP3 directly binds to ABCB1 mRNA in MDA-MB-231 and MDA-MB-231R cells, with stronger enrichment in resistant cells. ESR2 was used as a negative control, and MYC as a positive control, n = 4 in each group. ***P < 0.001. **(I)** RIP-qPCR showed that knockdown of METTL3 or METTL14 (core m^6^A methyltransferases) significantly reduced the binding of IGF2BP3 to ABCB1 mRNA in MDA-MB-231 cells, n = 4 in each group. ***P < 0.001. **(J)** mRNA decay assays (actinomycin D treatment) revealed that IGF2BP3 knockdown accelerated ABCB1 mRNA degradation in both MDA-MB-231 and MDA-MB-231R cells, n = 4 in each group.

VASN has not been previously reported to be involved in transcriptional regulation. However, VASN contains structural domains, which may enable it to participate in the transcriptional regulation of certain key drug-resistant molecules in TNBC by binding to downstream proteins. To elucidate the upstream regulatory mechanism of VASN in mediating paclitaxel resistance, we first identified VASN-interacting proteins in MDA-MB-231 cells using IP-MS (**Fig. 6A**). Among the high-scoring candidate proteins, insulin-like growth factor 2 binding protein 3 (IGF2BP3) was selected as a candidate for further validation, considering its well-documented roles in tumor progression and drug resistance. More importantly, previous studies have reported that IGF2BP3 mediates RNA methylation to participate in cellular transcriptional regulation. Subsequently, the results of Co-IP assays and immunofluorescence microscopy showed the direct physical interaction and colocalization of VASN and IGF2BP3 in cytoplasmic compartments (**Fig. 6B-C**). Additionally, computational prediction was conducted to identify the potential binding sites between VASN and IGF2BP3 **(Fig. 6D)**. Then, we investigated the effect of IGF2BP3 knockdown on ABCB1 expression and found that in MDA-MB-231 and CAL-51 cells, transfection with siIGF2BP3 significantly reduced both ABCB1 mRNA and protein levels **(Fig. 6E)**.

To verify whether ABCB1 mRNA carries m^6^A modifications, we performed m^6^A RNA immunoprecipitation quantitative polymerase chain reaction (m^6^A RIP-qPCR). Using EEF1A mRNA as a control (with its stop codon region used as a positive control and exon 5 as a negative control), we designed primers targeting a predicted m^6^A site in the coding sequence (CDS) region of ABCB1 mRNA (predicted by RMBase v2.0). As shown in **Fig. 6F**, the CDS region of ABCB1 mRNA exhibited significant m^6^A enrichment in both MDA-MB-231 and MDA-MB-231R cells, while there was no significant difference in m^6^A levels between parental and drug-resistant cells. This confirms that ABCB1 mRNA is modified by m^6^A in breast cancer cells. We next examined whether VASN directly regulates m6A modification of ABCB1 mRNA. The rescue experiments showed that VASN knockdown reduced m⁶A modification of ABCB1 mRNA in MDA-MB-231 cells, and this reduction was fully rescued by IGF2BP3 overexpression. Meanwhile, VASN overexpression alone had minimal impact on ABCB1 m⁶A levels, whereas IGF2BP3 silencing significantly decreased m⁶A enrichment regardless of VASN overexpression **(Fig. 6G)**. These results indicate that VASN does not directly regulate ABCB1 mRNA m⁶A modification, but acts through IGF2BP3.

Next, we performed RIP-qPCR to determine whether IGF2BP3 directly interacts with ABCB1 mRNA. Using ESR2 as a negative control and MYC as a positive control (a well-known target of IGF2BP3), we found that ABCB1 mRNA was significantly enriched in IGF2BP3 immunoprecipitates compared to immunoglobulin G (IgG) controls in both MDA-MB-231 and MDA-MB-231R cells (**Fig. 6H**). Notably, the enrichment of ABCB1 mRNA was more pronounced in drug-resistant MDA-MB-231R cells, indicating that the binding of IGF2BP3 to ABCB1 mRNA is dependent on the drug-resistant context.

Since METTL3 and METTL14 are core components of the m^6^A methyltransferase complex, we further explored whether m^6^A modification is necessary for the binding of IGF2BP3 to ABCB1 mRNA^20^, ^21^. Knockdown of METTL3 or METTL14 in MDA-MB-231 cells significantly impaired the interaction between IGF2BP3 and ABCB1 mRNA (**Fig. 6I**). To evaluate the effect of IGF2BP3 on ABCB1 mRNA stability, we performed mRNA decay assays using actinomycin D to block *de novo* transcription. In both MDA-MB-231 and MDA-MB-231R cells, IGF2BP3 knockdown significantly accelerated the degradation of ABCB1 mRNA (**Fig. 6J**).

Collectively, our findings reveal that VASN may upregulate the transcriptional level of ABCB1 via IGF2BP3-mediated m^6^A methylation of ABCB1 RNA, thereby promoting paclitaxel resistance in TNBC.

### VASN stabilizes IGF2BP3 protein by regulating K48-Linked polyubiquitination

**Figure 7:**
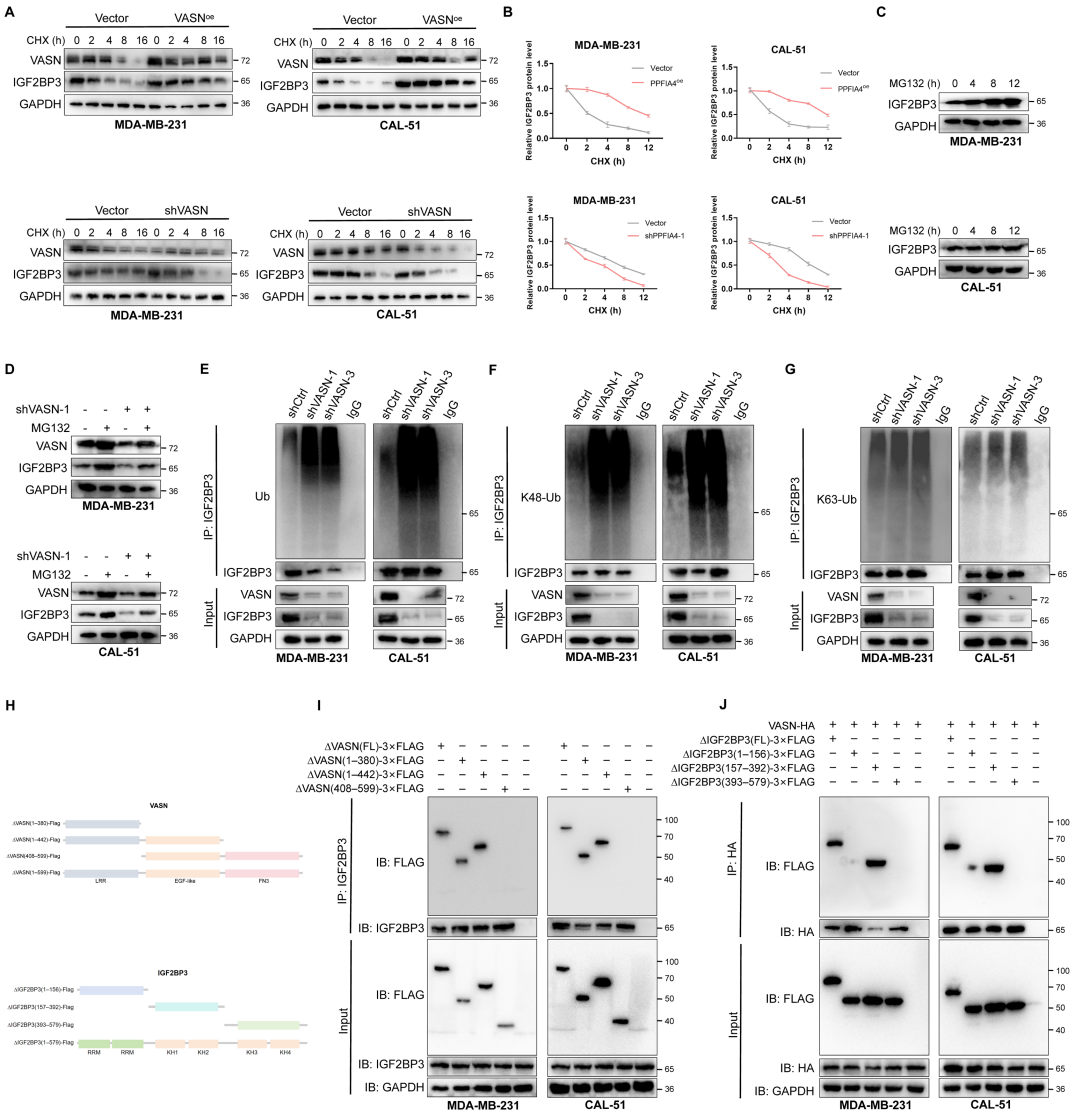
** VASN stabilizes IGF2BP3 protein by inhibiting its K48-linked polyubiquitination. (A, B)** Cycloheximide (CHX) chase assays showed that IGF2BP3 protein half-life was prolonged in VASN-overexpressing cells and shortened in VASN-knockdown (shVASN) MDA-MB-231 and CAL-51 cells, n = 3. **(C, D)** Western blot analysis demonstrated that proteasome inhibitor MG132, but not lysosome inhibitor bafilomycin A1, restored IGF2BP3 protein levels in shVASN cells, indicating proteasome-dependent degradation. **(E)** Ubiquitination-specific IP assays revealed that total ubiquitination of IGF2BP3 was increased in shVASN cells and reduced in VASN^oe^ cells. **(F, G)** IP assays using K48- and K63-specific ubiquitin antibodies showed that VASN depletion specifically enhanced K48-linked polyubiquitination of IGF2BP3, while VASN overexpression inhibited this modification. **(H)** Schematic representation of VASN and IGF2BP3 truncation mutants used for domain mapping. **(I, J)** Co-IP assays with truncated mutants revealed that VASN interacts with IGF2BP3 via its LRR domain, while IGF2BP3 utilizes its KH domain for this association.

Given our preliminary findings that VASN had no effect on IGF2BP3 transcriptional levels, we hypothesized that VASN might regulate IGF2BP3 protein abundance at the post-translational level, most likely through modulation of protein degradation (**Fig. S6A**). Subsequently, IGF2BP3 protein stability was assessed via cycloheximide (CHX) chase experiments in control, VASN-overexpressing, and VASN-knockdown MDA-MB-231 and CAL-51 cells. The half-life of IGF2BP3 was prolonged in VASN^oe^ cells and shortened in shVASN cells (**Fig. 7A-B**), suggesting VASN enhances IGF2BP3 protein stability. We further found that treatment with the proteasome inhibitor MG132, but not the lysosome inhibitor bafilomycin A1, restored IGF2BP3 levels in shVASN cells, indicating VASN regulates IGF2BP3 degradation in a proteasome-dependent manner (**Fig. 7C-D; Fig. S6B**).

Considering that VASN modulates IGF2BP3 protein degradation, we then determined whether this regulatory effect is mediated by the modulation of IGF2BP3 ubiquitination status^22^, ^23^. Ubiquitination-specific IP assays demonstrated that total ubiquitination of IGF2BP3 was markedly increased in VASN-knockdown (shVASN) cells, whereas it was significantly reduced in VASN-overexpressing (VASN-OE) cells (**Fig. 7E; Fig. S6C**). Using antibodies specific to K48- and K63-linked polyubiquitin chains, we further found that VASN depletion robustly enhanced both K48-linked polyubiquitination of IGF2BP3, while VASN overexpression conversely inhibited this type of polyubiquitination modifications (**Fig. 7F-G; Fig. S6D-E**). We further identified the specific domains mediating their association, truncation mutant interaction assays were conducted. We generated truncated constructs of VASN and IGF2BP3 (**Fig. 7H**). To pinpoint the binding regions, we generated truncated mutants of both proteins and performed Co-IP assays. These experiments revealed that VASN interacts with IGF2BP3 via its LRR domain, while IGF2BP3 utilizes its KH domain for this association (**Fig. 7I-J**).

To translate our *in vitro* mechanistic insights into clinical relevance, we performed IHC staining to detect the protein expression of VASN, IGF2BP3 and ABCB1 in clinical TNBC tissue specimens, with systematic quantitative analysis of IHC staining intensity conducted (**Fig. S6F**). Kaplan-Meier survival analysis with the log-rank test confirmed that TNBC patients with high VASN/IGF2BP3/ABCB1 expression had a significantly shorter disease-free survival DFS (**Fig. S6G**). Additionally, correlation analysis demonstrated that high expression of VASN, IGF2BP3 and ABCB1 was positively correlated with advanced tumor stage in TNBC patients (**Fig. S6H**). Furthermore, elevated expression of these three molecules was significantly associated with chemoresistance in TNBC patients (**Fig. S6I**). To identify independent predictors of pCR to neoadjuvant chemotherapy in TNBC patients, we collected complete clinicopathological data and performed univariate and multivariate logistic regression analyses. Multivariate analysis revealed that N stage (P = 0.036), Ki-67 index (P < 0.001), and VASN expression level (P = 0.012) were independent factors significantly associated with pCR after NACT **(Table S8)**.

Collectively, these findings demonstrate that VASN interacts with IGF2BP3 to suppress its K48-linked polyubiquitination, thereby inhibiting proteasomal degradation of IGF2BP3. Stabilized IGF2BP3 further promotes ABCB1 transcription, ultimately contributing to chemoresistance in breast cancer cells.

### VASN recruits USP10 to stabilize IGF2BP3 by attenuating K48-Linked polyubiquitination

**Figure 8:**
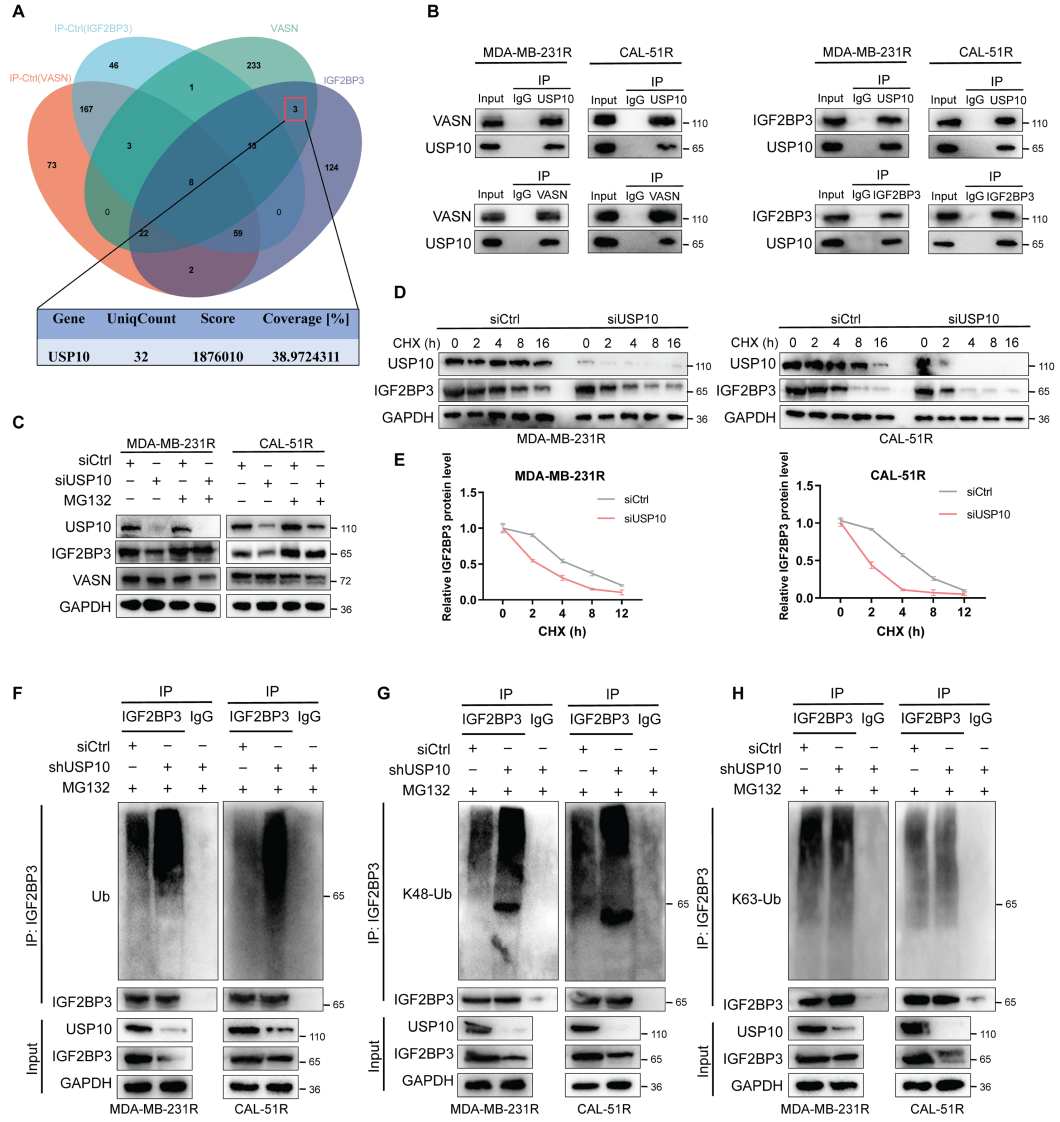
** VASN recruits USP10 to stabilize IGF2BP3 by attenuating K48-linked polyubiquitination. (A)** Venn diagram of VASN and IGF2BP3 interactomes, identifying ubiquitin-specific protease 10 (USP10) as the top candidate deubiquitinating enzyme (DUB). The table below shows the key metrics for USP10 from the IP-MS analysis. **(B)** Co-IP assays confirmed that USP10 directly interacts with both VASN and IGF2BP3 in MDA-MB-231R and CAL-51R cells, supporting the formation of a VASN-USP10-IGF2BP3 ternary complex. **(C)** Western blot analysis showed that USP10 knockdown (siUSP10) reduced IGF2BP3 protein levels, and this reduction was rescued by treatment with the proteasome inhibitor MG132, indicating proteasome-dependent degradation. **(D, E)** Cycloheximide (CHX) chase assays demonstrated that USP10 knockdown significantly shortened the half-life of IGF2BP3 in MDA-MB-231R and CAL-51R cells, n = 3. **(F)** Ubiquitination-specific IP assays revealed that USP10 depletion increased the total ubiquitination of IGF2BP3. **(G, H)** IP assays using K48- and K63-specific ubiquitin antibodies showed that USP10 knockdown selectively enhanced K48-linked polyubiquitination of IGF2BP3, while K63-linked polyubiquitination remained unchanged.

Building on our previous findings that VASN enhances IGF2BP3 protein stability, we hypothesized that this regulatory effect might be mediated by recruiting deubiquitinating enzymes (DUBs) to counteract IGF2BP3 ubiquitination^24^. To identify the relevant DUB, we performed an integrative analysis of VASN and IGF2BP3 interactome datasets, which identified ubiquitin-specific protease 10 (USP10) as the top candidate DUB (**Fig. 8A**).

Co-IP assays confirmed that USP10 directly interacts with both VASN and IGF2BP3 in MDA-MB-231R and CAL-51R cells, supporting the formation of a VASN-USP10-IGF2BP3 ternary complex (**Fig. 8B**). We next investigated the functional impact of USP10 on IGF2BP3 protein homeostasis. Depletion of USP10 via siRNA significantly reduced IGF2BP3 protein levels, and this reduction was rescued by treatment with the proteasome inhibitor MG132, indicating USP10 regulates IGF2BP3 stability in a proteasome-dependent manner (**Fig. 8C**).

CHX chase experiments further demonstrated that USP10 knockdown markedly shortened the half-life of IGF2BP3 in both MDA-MB-231R and CAL-51R cells (**Fig. 8D-E**), phenocopying the destabilization effect of VASN depletion. To confirm that USP10 acts through deubiquitination, we performed ubiquitination-specific IP assays. USP10 depletion increased total ubiquitination of IGF2BP3 (**Fig. 8F**). Further analysis with K48- and K63-linked ubiquitin chain-specific antibodies revealed that USP10 knockdown selectively enhanced K48-linked polyubiquitination of IGF2BP3, whereas no significant change in K63-linked polyubiquitination was observed (**Fig. 8G-H**).

In conclusion, our study revealed a mechanistic pathway where VASN recruits the DUB USP10 to deubiquitinate IGF2BP3, specifically reducing K48- and K63-linked polyubiquitination, thereby antagonizing proteasomal degradation and stabilizing IGF2BP3 protein in triple-negative breast cancer cells.

### Targeting VASN by trametinib enables synergistic paclitaxel therapy against chemoresistant TNBC

**Figure 9:**
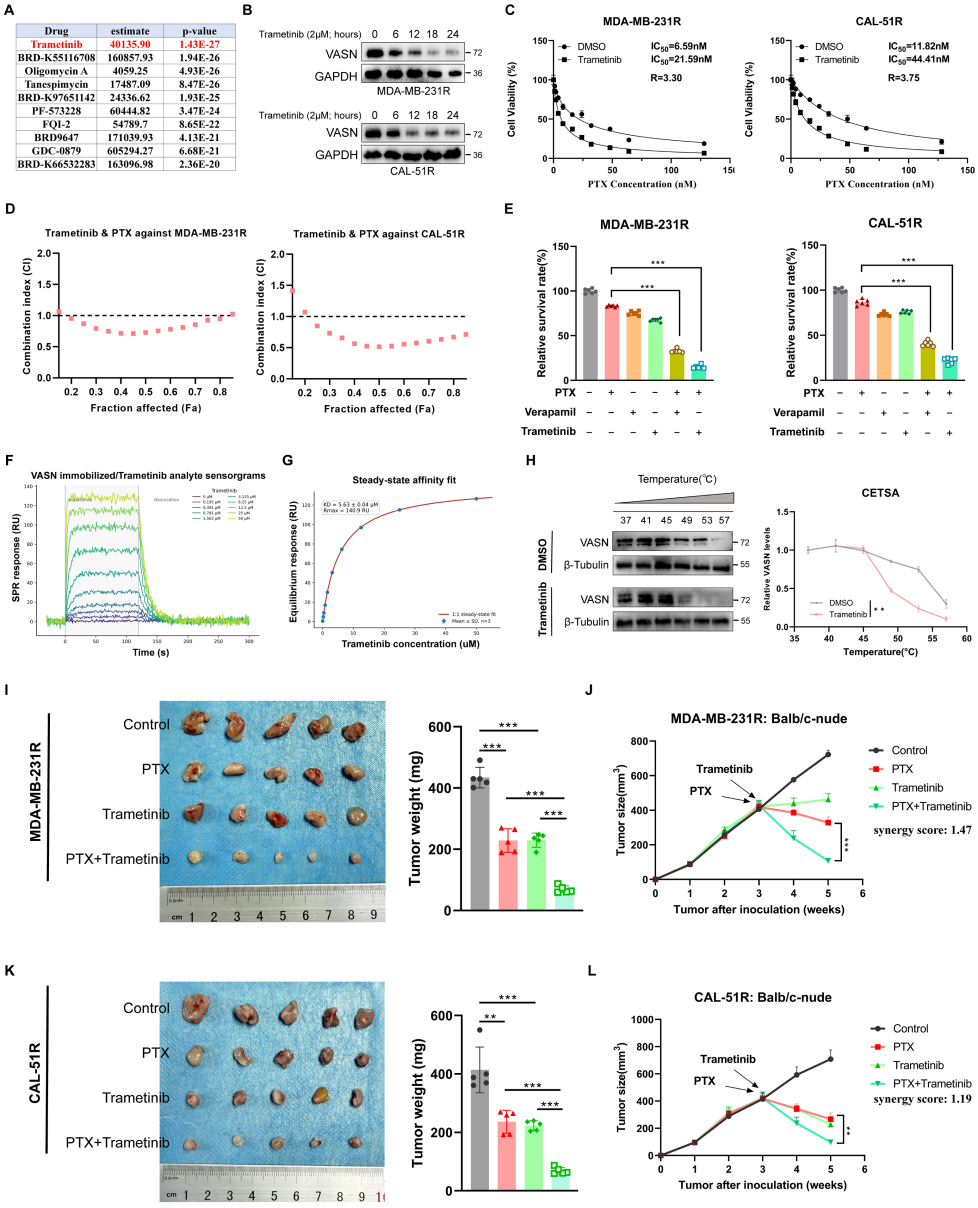
** Trametinib targets VASN to restore paclitaxel sensitivity in TNBC *in vitro* and *in vivo*. (A)**Drug screening results using the oncoPredict R package and CTRPv2 dataset, identifying Trametinib as a top candidate compound targeting VASN-mediated paclitaxel resistance. **(D)** Western blot analysis demonstrating time-dependent depletion of VASN protein in MDA-MB-231R and CAL-51R cells following Trametinib treatment (2 μM). **(C)** Cell viability assays showing that Trametinib sensitized MDA-MB-231R and CAL-51R cells to paclitaxel, with reduced IC₅₀ values for paclitaxel in the presence of Trametinib, n = 3. **(D)** Chou-Talalay analysis of the combination effect of Trametinib and paclitaxel in MDA-MB-231R and CAL-51R cells, n = 3. **(E)** Relative cell survival rates of MDA-MB-231R and CAL-51R cells treated with paclitaxel, Trametinib, verapamil (ABCB1 inhibitor), or their combinations. Trametinib synergistically enhanced paclitaxel sensitivity, n = 5. **P < 0.01, ***P < 0.001. **(F, G)** Surface plasmon resonance (SPR) analysis of the direct interaction between VASN protein and Trametinib. Representative sensorgrams showing the binding kinetics of Trametinib (0-50 μM) to immobilized recombinant VASN protein **(F)**. Steady-state affinity fitting using a 1:1 binding model **(G)**. **(H)** Cellular thermal shift assay (CETSA) assay of VASN stability in DMSO and Trametinib-treated cells. (Left) Representative immunoblots comparing VASN thermal stability in cells treated with DMSO or Trametinib at various temperatures (37-57 °C). (Right) Quantified decay curves of VASN protein across temperatures, n = 3. Student's t-test, **p < 0.01.**(I)** Representative images of excised tumors from BALB/c-nude mice bearing MDA-MB-231R orthotopic xenografts following treatment with control, paclitaxel, Trametinib, or paclitaxel+Trametinib. And quantification of final tumor weights from MDA-MB-231R xenografts, confirming the superior efficacy of the combination therapy, n = 5 in each group. **P < 0.01, ***P < 0.001.** (J)** *In vivo* tumor growth curves of MDA-MB-231R xenografts, showing that the combination of paclitaxel and Trametinib produced a synergistic inhibitory effect on tumor growth, n = 5 in each group. The synergy score was calculated using the Bliss independence model. **(K)** Representative images of excised tumors from BALB/c-nude mice bearing CAL-51R orthotopic xenografts following treatment with control, paclitaxel, Trametinib, or paclitaxel+Trametinib. And quantification of final tumor weights from CAL-51R xenografts, confirming the superior efficacy of the combination therapy, n = 5 in each group. **P < 0.01, ***P < 0.001.** (L)** *In vivo* tumor growth curves of CAL-51R xenografts, showing that the combination of paclitaxel and Trametinib produced a synergistic inhibitory effect on tumor growth. The synergy score was calculated using the Bliss independence model, n = 5 in each group.

This study screened VASN-associated therapeutic drugs using the oncoPredict R package and CTRPv2 dataset (**Fig. 9A**). Trametinib was selected as the lead compound for functional validation of targeting VASN-mediated PTX resistance in TNBC. The chemical structure of Trametinib and its molecular docking model with VASN were visualized, demonstrating stable binding via hydrogen bonding and hydrophobic interactions (**Fig. S7A**). qRT-PCR analysis showed that Trametinib treatment did not significantly alter VASN mRNA expression in MDA-MB-231R and CAL-51R cells within 24 hours (**Fig. S7B**). WB analysis revealed that Trametinib induced time-dependent depletion of VASN protein in MDA-MB-231R and CAL-51R cells (**Fig. 9B**). In MDA-MB-231R and CAL-51R cells, the apoptosis marker cleaved caspase-3 was significantly upregulated under the combined treatment of trastuzumab and PTX compared to PTX alone (**Fig. S7C**). Cell viability assays showed that Trametinib sensitized MDA-MB-231R and CAL-51R cells to paclitaxel, with reduced IC_50_ values for paclitaxel in the presence of Trametinib (**Fig. 9C**).

To further understand the synergistic effect between trametinib and paclitaxel, we performed Chou-Talalay assays in MDA-MB-231R and CAL-51R cells. For MDA-MB-231R cells, CI values were all less than 1 at Fa 0.2-0.8, indicating a synergistic effect between the two drugs within this range of inhibition rates. Similarly, for CAL-51R cells, CI values were all less than 1 at Fa 0.25-0.85, confirming a strong synergistic effect between trametinib and paclitaxel (**Fig. 9D**). Cell survival assays demonstrated that Trametinib synergized with paclitaxel to suppress MDA-MB-231R and CAL-51R cell viability, with combination treatments showing greater efficacy than monotherapies (**Fig. 9E**). Colony formation assays confirmed that co-treatment with Trametinib and paclitaxel reduced the clonogenic potential of MDA-MB-231R and CAL-51R cells more profoundly than single agents (**Fig. S7D**).

Subsequently, we detected whether VASN is a direct target of Trametinib. We performed SPR analysis by immobilizing recombinant VASN protein on a sensor chip and flowing Trametinib at gradient concentrations ranging from 0 to 50 μM. Trametinib bound to immobilized VASN in a clear dose-dependent manner, with a fitted equilibrium dissociation constant (KD) of 5.63 ± 0.04 μM. This result unequivocally validates a direct, physical interaction between Trametinib and VASN (**Fig. 9F-G**). In addition, to identified the stability of the complex formed by the VASN protein and trametinib. The system underwent pronounced inward contraction and conformational optimization during the first 20 ns of the simulation, followed by a highly stable plateau from 20 to 100 ns, during which the RMSD remained around 1.8 nm with minimal fluctuations, indicating that the complex maintained a compact and rigid native-like fold in aqueous solution (**Fig. S7E**). The free energy landscape (FEL) displayed a concentrated, deep energy basin with well-defined boundaries, confirming that the system predominantly resides in the thermodynamically most favorable conformation (**Fig. S7F**). Furthermore, we performed CETSA to evaluate whether Trametinib alters the thermal stability of the VASN protein. First, using a broad range of concentration gradients, we assessed the degradation of purified VASN protein, with the more thermally stable β-Tubulin protein used as a control (**Fig. S7G**). The results showed that VASN protein underwent pronounced degradation at approximately 56 °C. Therefore, starting from human body temperature (37 °C), we performed a gradient analysis of the effect of Trametinib on VASN thermal stability. The results showed that treatment with Trametinib led to a clear reduction in VASN thermal stability, as evidenced by a leftward shift of the melting curve compared with the vehicle control. These experimental results indicate that Trametinib can bind to purified VASN protein *in vitro* and affect the thermal stability of the VASN protein (**Fig. 9H**). Furthermore, the result of Co-IP assay demonstrated that after treatment with Trametinib, the binding between IGF2BP3 and USP10 was significantly reduced (**Fig. S7H**).

Orthotopic xenograft models were established in BALB/C-nude mice using MDA-MB-231R and CAL-51R cells to evaluate *in vivo* efficacy. Analysis of representative tumor images and measurements revealed that both paclitaxel and trametinib monotherapies inhibited tumor growth, while their combination produced a synergistic effect, leading to a more substantial reduction in tumor burden (**Fig. 9I-L**). IHC analyses for Ki-67 revealed that the combination treatment group exhibited a significant reduction in cell proliferation (**Fig. S7I**). Furthermore, no significant difference in body weight was observed among the treatment groups (P > 0.05) (**Fig. S7J**). Measurement of serum lactate dehydrogenase (LDH), creatine kinase-MB (CK-MB), and creatinine levels indicated that the combination therapy did not induce significant damage to hepatic, cardiac, or renal function (**Fig. S7K-M**). HE staining of major organs demonstrated well-preserved histological structure in the heart, liver, and kidneys following treatments (**Fig. S7N**).

Therefore, our screening and validation experiments demonstrate that Trametinib effectively suppresses VASN protein expression. Furthermore, when combined with paclitaxel, it exhibits a synergistic therapeutic effect against TNBC, accompanied by a favorable biosafety profile.

## Discussion

**Figure 10:**
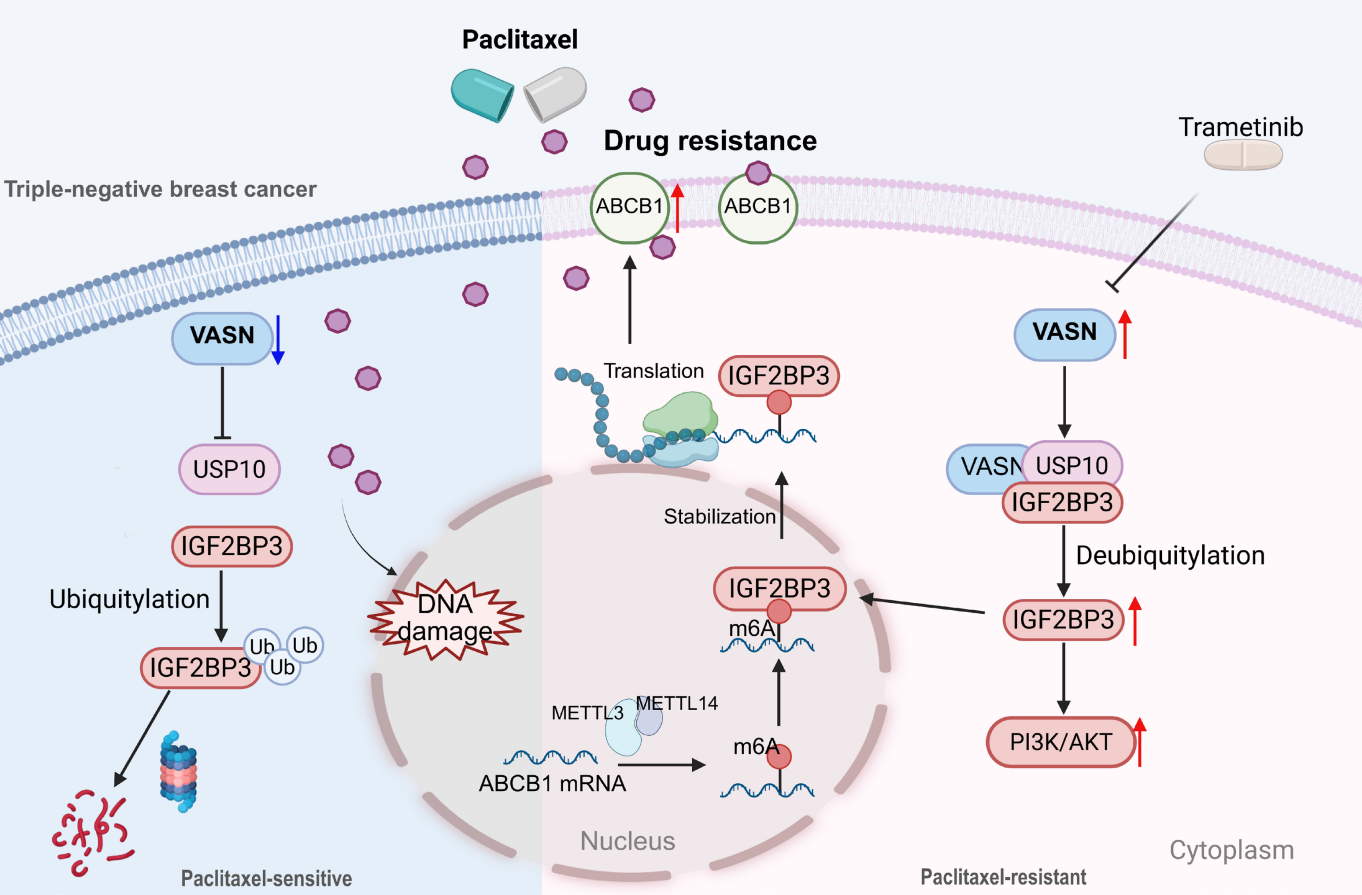
** Graphic abstract of this article.** Schematic model of VASN-mediated paclitaxel resistance in TNBC. In VASN high TNBC cells, VASN recruits USP10 to form a ternary complex with IGF2BP3, promoting IGF2BP3 protein deubiquitination and stabilization. Elevated IGF2BP3 activates the PI3K/AKT pathway and translocates into the nucleus to modulate m^6^A methylation and upregulating ABCB1 expression. Increased paclitaxel efflux and activation of anti-apoptotic signaling lead TNBC paclitaxel resistance. In contrast, VASN low-expression or targeting VASN via Trametinib abrogates USP10 recruitment, enhancing IGF2BP3 ubiquitination and degradation. This disrupts downstream signaling, restoring TNBC cell sensitivity to paclitaxel.

Paclitaxel-based chemotherapy is a first-line treatment modality for TNBC, yet the development of acquired chemoresistance markedly compromises its clinical therapeutic efficacy. To identify potential targets for clinical intervention, we established paclitaxel-resistant TNBC cell lines (MDA-MB-231R and CAL-51R) and collected clinical breast cancer specimens. Using these preclinical and clinical models, we identified VASN as a critical regulator of paclitaxel resistance in TNBC. Mechanistically, we demonstrated that VASN is transcriptionally regulated upstream by CEBPB and orchestrates the resistant phenotype via a comprehensive pathway. This finding not only provides novel insights into the molecular mechanisms underlying chemoresistance in TNBC but also fills a gap in the understanding of VASN in the field of tumor drug resistance. Furthermore, it expands the research dimension concerning m^6^A methylation modification and its regulatory network with paclitaxel resistance-related genes (**Figure 10**). The VASN-associated pathway and combination therapeutic strategy identified in this study offer potential targets and therapeutic perspectives for addressing this clinical challenge.

Through transcriptome sequencing analysis of paclitaxel-resistant cell lines and clinical samples from patients with TNBC treated at our institution, this study identified a series of potential key regulatory genes associated with paclitaxel resistance. Among these, genes such as RASGRP1 and HMGCS1 have been previously documented to be involved in paclitaxel resistance. Vasorin (VASN), a ubiquitously expressed glycoprotein conserved in humans and other animals, has three distinct isoforms, including a transmembrane isoform, a secreted variant (sVASN), and an intracellular isoform^25^, ^26^. The extracellular secretion of sVASN is generated by proteolytic cleavage of transmembrane VASN, and this soluble protein is capable of suppressing the TGF-β signaling pathway. This mechanism may promote tumor immune evasion or metastasis within the tumor microenvironment (TME) by relieving TGF-β-mediated suppression of tumor growth. In breast cancer, specific O-glycosylation modifications of sVASN, particularly sialylation events mediated by the enzyme ST3Gal1, enhance its binding affinity to TGF-β1^27^.

Meanwhile, the intracellular and transmembrane isoforms of VASN are also capable of activating oncogenic signaling pathways to promote tumorigenesis and progression. In laryngeal and prostate cancers, VASN facilitates tumor progression through modulating the expression of YAP/TAZ oncoproteins^28^. Accumulating evidence has demonstrated that VASN exerts its pro-tumor functions via physical interaction with specific target proteins, which in turn activates the biological functions of these proteins and their downstream signaling cascades. For instance, VASN binds to the NOTCH1 receptor to trigger the activation of both NOTCH and MAPK signaling pathways, thereby enhancing cancer cell metastasis and chemoresistance in colorectal cancer (CRC)^10^. In gastric cancer, VASN drives tumor initiation and progression by regulating COL4A1 expression and subsequently activating the PI3K/AKT oncogenic pathway^11^. In glioma, VASN directly interacts with vascular endothelial growth factor receptor 2 (VEGFR2) and induces its autophosphorylation, which further activates the AKT signaling cascade to accelerate tumor angiogenesis^29^. In this study, IP-MS assay was employed to screen and validate that VASN forms a complex with the deubiquitinase USP10 and IGF2BP3 in cells. This interaction diminishes the ubiquitination level of IGF2BP3, thereby inhibiting its proteasomal degradation and increasing its protein stability, which represents a critical upstream regulatory mechanism driving chemoresistance in cancer cells. The ubiquitin-proteasome system serves as the central pathway for protein degradation in eukaryotes^30^, ^31^. Previous studies have demonstrated that USP10 diminishes the ubiquitination level of IGF2BP3, a finding that is consistent with our observations^32^. Furthermore, we extended these findings by showing that knockdown of VASN in TNBC cells led to a concomitant reduction in the interaction between USP10 and IGF2BP3, thus establishing our work as an in-depth exploration of the regulatory network underlying the prior discoveries.

Analysis of VASN downstream genes revealed that overexpression of VASN upregulates multiple genes and activates several pathways associated with paclitaxel resistance. Among these, the PI3K/AKT pathway was identified as a major activated pathway, which is closely linked to anti-apoptotic processes and tumor progression^33^. Moreover, experimental activation or suppression of this pathway could partially rescue paclitaxel sensitivity altered by changes in VASN expression. Notably, ABCB1 was identified among the genes showing the most pronounced expression changes. ABCB1 (P-glycoprotein) has been extensively reported as a key mediator of paclitaxel resistance^34^. For instance, in paclitaxel-resistant pancreatic ductal adenocarcinoma (PDAC) models, amplification of the ABCB1 genomic locus leads to its upregulation, representing a core mechanism of resistance^35^. This upregulation is considered a specific feature of resistant PDAC cells. Similarly, in non-small cell lung cancer (NSCLC) resistant cell lines, ABCB1 overexpression is regulated through the HNF1A/SHH signaling axis, thereby promoting drug resistance^36^. Functionally, ABCB1-encoded P-glycoprotein acts as an efflux pump in tumor cells, utilizing ATP hydrolysis to actively expel paclitaxel from cells, thereby reducing intracellular drug concentration^37^. Experimental evidence demonstrates that ABCB1 inhibitors (e.g., verapamil) or siRNA-mediated knockdown of ABCB1 significantly increase intracellular accumulation of paclitaxel and reverse resistance^34^, ^38^, ^39^. In our study, we further confirmed that ABCB1, as a downstream gene of VASN, contributes to paclitaxel resistance in TNBC. Targeting ABCB1 function or its upstream signaling pathways represents a promising clinical strategy to reverse paclitaxel resistance and enhance therapeutic efficacy.

Previous studies have revealed that IGF2BP3 can drive multidrug resistance in colorectal cancer by regulating ABCB1 expression through an m⁶A-dependent mechanism^40^. Our study further confirms through RIP and m⁶A immunoprecipitation assays that a similar regulatory axis exists in TNBC. In this study, VASN-knockdown significantly downregulated both mRNA and protein levels of ABCB1, whereas overexpression of VASN upregulated ABCB1 expression. Moreover, knockdown of ABCB1 reversed the VASN-mediated resistant phenotype, directly validating ABCB1 as a key downstream effector in the VASN pathway. As the most prevalent epitranscriptomic modification on eukaryotic mRNA, m⁶A methylation was further investigated in this context. We found that silencing the m⁶A methyltransferase complex METTL3/METTL14 markedly reduced both the m⁶A modification level and expression of ABCB1. This suggests that IGF2BP3 may recruit the METTL3/METTL14 complex to catalyze m⁶A modification on ABCB1 mRNA, thereby enhancing its mRNA stability or facilitating mRNA maturation and translation. This finding establishes a link between m⁶A epitranscriptomic modification and the classic ABCB1-mediated multidrug resistance pathway, clarifies the specific molecular mechanism by which IGF2BP3, as an m⁶A reader, regulates ABCB1 expression, reveals a novel epigenetic regulatory mechanism underlying resistance in TNBC, and further underscores the multifaceted role of VASN as a critical regulator of drug resistance.

More importantly, we collected hundreds of TNBC patient samples and performed IHC staining for the three key genes in our study. All three genes were found to be highly expressed in paclitaxel-resistant tissues, and their expression was strongly associated with advanced disease stage and poor prognosis. This underscores the significant clinical relevance of these genes in identifying TNBC patients with paclitaxel resistance. As previously reported, IGF2BP3 is significantly upregulated in breast cancer tissues, and this differential expression positions it as a potential diagnostic biomarker. Its expression is notably higher in the TNBC subtype, demonstrating a sensitivity of 38.0% and a specificity of 98.2% for predicting TNBC. High IGF2BP3 expression is significantly correlated with higher tumor grade and elevated Ki-67 index, indicating its association with a more aggressive phenotype^41^. Multivariate analysis has confirmed IGF2BP3 as an independent risk factor for distant metastasis-free survival (HR = 1.876)^42^. Furthermore, IGF2BP3 promotes breast cancer cell proliferation and drug resistance by binding to CD44 mRNA, which stimulates IGF2 expression in fibroblasts and activates the Hedgehog signaling pathway^43^. Similarly, assessing ABCB1 expression levels holds substantial clinical value for predicting paclitaxel sensitivity in malignant tumors^44^. Our study innovatively identifies the driving role of VASN in chemotherapy resistance in TNBC and establishes its correlation with poor patient prognosis. Finally, by screening the FDA drug database, we identified Trametinib as a compound capable of binding to a critical region of the VASN protein, thereby downregulating VASN expression and reversing paclitaxel resistance in TNBC. This finding offers a promising potential combination therapy for clinical patients who have developed resistance.

While this study elucidates the core regulatory pathway and proposes a potential therapeutic strategy, several limitations warrant consideration. First, the research was primarily conducted using the MDA-MB-231 and CAL-51 cell lines, which may not fully capture the heterogeneity of paclitaxel resistance across all TNBC subtypes. Second, the precise mechanistic role of USP10 within this pathway requires further elucidation. Specifically, how VASN regulates the deubiquitinating activity of USP10, and whether their interaction influences USP10's regulation of other substrates, has yet to be fully explored. Finally, this study primarily focused on ABCB1 as a key downstream target. Given that chemoresistance in TNBC is orchestrated by multiple genes and pathways, it remains to be determined whether the VASN-IGF2BP3 axis regulates other m^6^A-modified, resistance-associated genes.

In summary, our study delineates a mechanistic framework for VASN-dependent paclitaxel resistance in TNBC. We demonstrate that resistance is mediated via the VASN/USP10/IGF2BP3-ABCB1 axis, thereby highlighting the potential of VASN as both a prognostic biomarker and a therapeutic target. The synergistic effect observed with the combination of Trametinib and paclitaxel offers a precise therapeutic strategy to combat this heterogeneous disease, particularly in patients with established resistance.

## Supplementary Material

Supplementary figures and tables.

## Data Availability

All raw data supporting the findings of this study can be obtained from the corresponding author upon reasonable request. Furthermore, the transcriptomic data presented herein have been submitted to the GenBase repository, hosted by the National Genomics Data Center (NGDC), China National Center for Bioinformation / Beijing Institute of Genomics, Chinese Academy of Sciences. These data are openly available at https://ngdc.cncb.ac.cn/genbase under the accession code CRA039413.
