# Supplementary material for: VASN Enhances IGF2BP3 Stability via USP10 Deubiquitination to Promote Triple-negative Breast Cancer Paclitaxel Resistance
Source: Int J Biol Sci. 2026 Jul 13;22(12):6583–607. doi: 10.7150/ijbs.136403 (PMC13411843; doi:10.7150/ijbs.136403)
Supplement: Supplementary file 1 — Supplementary figures and tables. [file ijbsv22p6583s1.pdf]

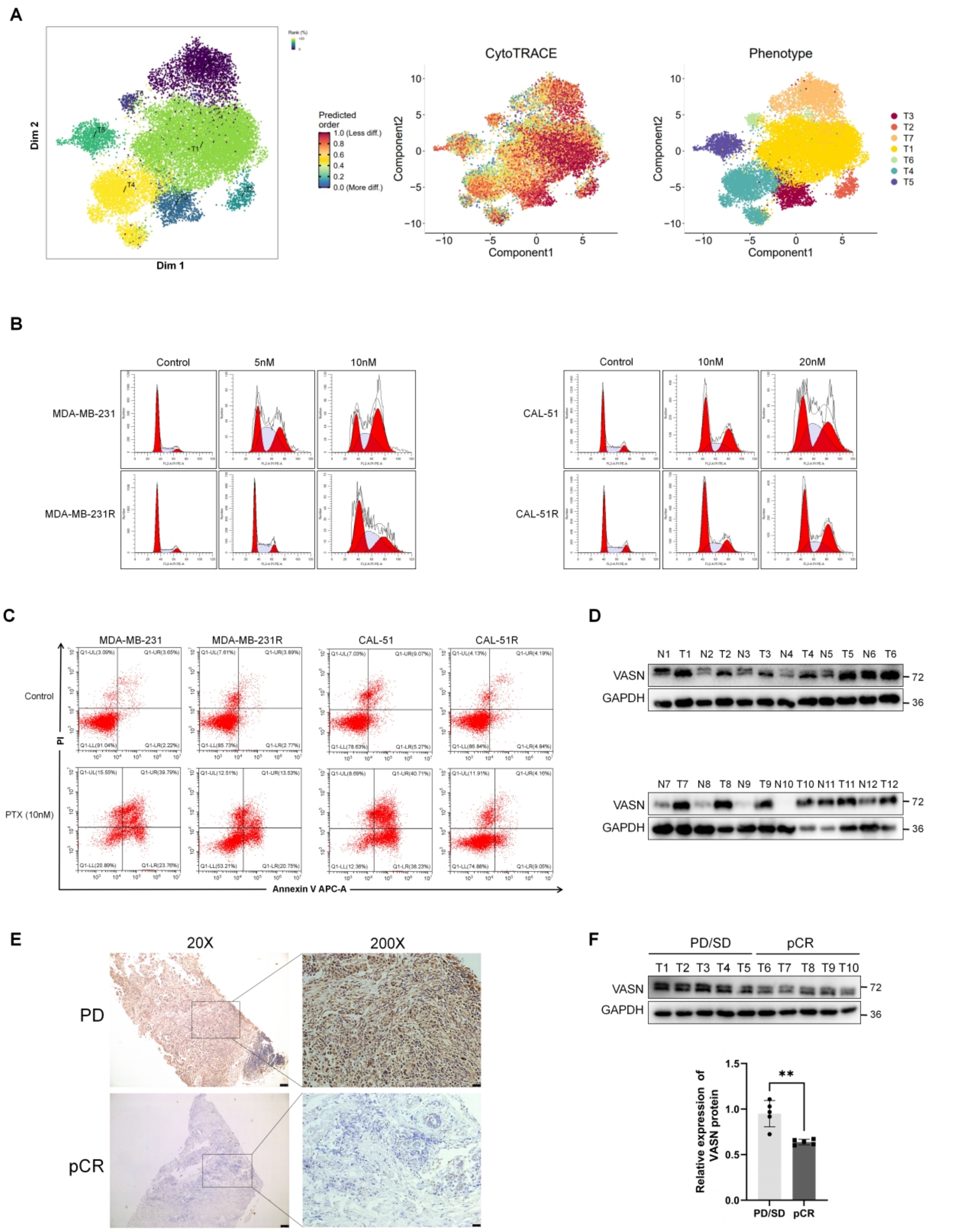

Figure S1. High expression of VASN in paclitaxel-resistant TNBC

(A)UMAP visualization of scRNA-seq data (GSE169246) from TNBC patient samples, colored by cell type (left) and by CytoTRACE score (middle) to infer cell differentiation status, and colored by phenotypic clusters (right) to show subpopulation structure. **(B)** Flow cytometry histograms of cell cycle distribution in MDA-MB-231, MDA-MB-231R, CAL-51, and CAL-51R cells treated with paclitaxel at 5 nM or 10 nM for 24 h. **(C)** Flow cytometry dot plots of apoptosis in parental and paclitaxel-resistant TNBC sublines treated with paclitaxel (10 nM) for 48 h, with quantification of Annexin V-positive apoptotic cells. **(D)** Western blot analysis of VASN protein expression in paired paracancerous (N) and tumor (T) tissues from 16 TNBC patients, with

GAPDH as a loading control. **(E)** Representative IHC images of VASN expression in TNBC patient tissues stratified by treatment response. **(F)** Western blot analysis of VASN protein expression in TNBC patients stratified by response to paclitaxel-based therapy with quantification of relative VASN expression normalized to GAPDH. *$P < 0.05$.

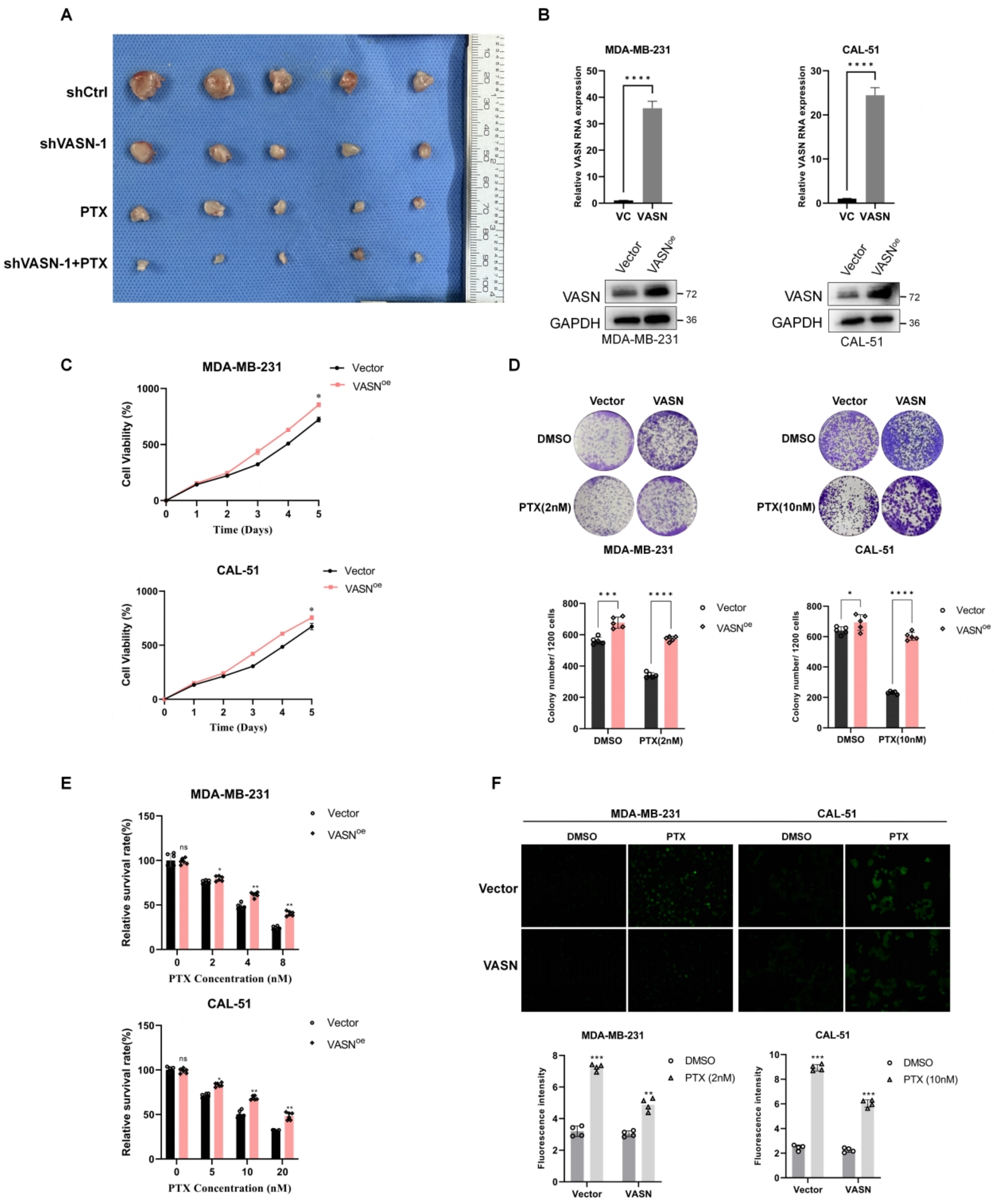

Figure S2. VASN overexpression enhances TNBC cell proliferation and paclitaxel resistance

(A) Representative images of excised tumors from nude mice bearing MDA-MB-231 xenografts following treatment with shCtrl, shVASN-1, paclitaxel, or the combination of shVASN-1 and PTX. **(B)** Validation of VASN overexpression efficiency in MDA-MB-231 and CAL-51 cells transfected with empty vector or VASN-overexpressing plasmid at the mRNA (top) and protein (bottom) levels. GAPDH served as a loading control. ****P < 0.0001. **(C)** Cell proliferation curves of vector-control and VASN-overexpressing MDA-MB-231 and CAL-51 cells over a 5-day period, demonstrating accelerated growth in VASN-overexpressing cells. **(D)** Representative colony formation images (top) and quantification (bottom) of vector-control and VASN-overexpressing TNBC cells treated with DMSO or paclitaxel. VASN overexpression rescued the paclitaxel-induced reduction in colony formation ability. **P < 0.01, ***P < 0.001, ****P <

0.0001. **(E)** Relative survival rates of vector-control and VASN-overexpressing MDA-MB-231 and CAL-51 cells treated with increasing concentrations of paclitaxel. **P < 0.01, ***P < 0.001. **(F)** Representative fluorescence microscopy images (top) and quantification of relative fluorescence intensity (bottom) of ROS levels in vector-control and VASN-overexpressing MDA-MB-231 and CAL-51 cells treated with DMSO or paclitaxel. ***P < 0.001.

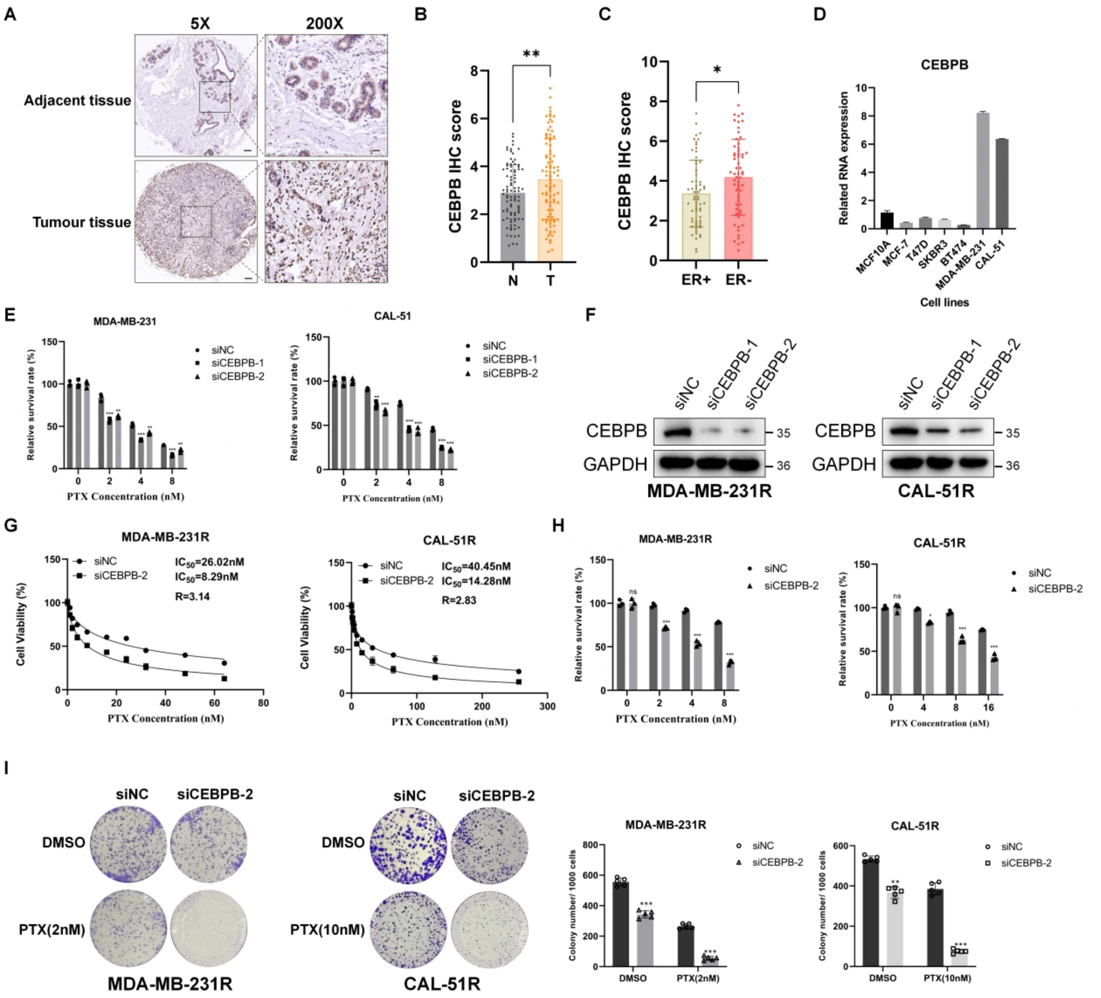

Figure S3. CEBPB is a key transcription factor of VASN

(A) Representative IHC images of CEBPB expression in paired adjacent normal and TNBC tumor tissues, shown at 5× and 200× magnification. **(B, C)** Quantification of CEBPB IHC scores in normal (N) vs. tumor (T) breast tissues (B) and in ER-positive (ER+) vs. ER-negative (ER−) breast cancer subtypes (C). **P < 0.05, **P < 0.01. **(D)** CEBPB mRNA expression in a panel of breast cell lines. **(E)** Relative survival rates of parental MDA-MB-231 and CAL-51 cells transfected with control siRNA (siNC) or CEBPB siRNAs (siCEBPB-1, siCEBPB-2) and treated with increasing concentrations of paclitaxel. **(F)** Western blot validation of CEBPB knockdown efficiency in paclitaxel-resistant MDA-MB-231R and CAL-51R cells transfected with siNC, siCEBPB-1, or siCEBPB-2. GAPDH was used as a loading control. **(G)** Cell viability assays of MDA-MB-231R and CAL-51R cells transfected with siNC or siCEBPB-2 and treated with increasing concentrations of paclitaxel. **(H)** Relative survival rates of MDA-MB-231R and CAL-51R cells transfected with siNC or siCEBPB-2 and treated with increasing concentrations of paclitaxel; *P < 0.05, **P < 0.01. **(I)** Representative colony formation images (left) and quantification (right) of MDA-MB-231R and CAL-51R cells transfected with siNC or siCEBPB-2 and treated with DMSO or paclitaxel. ***P < 0.001, ****P < 0.0001.

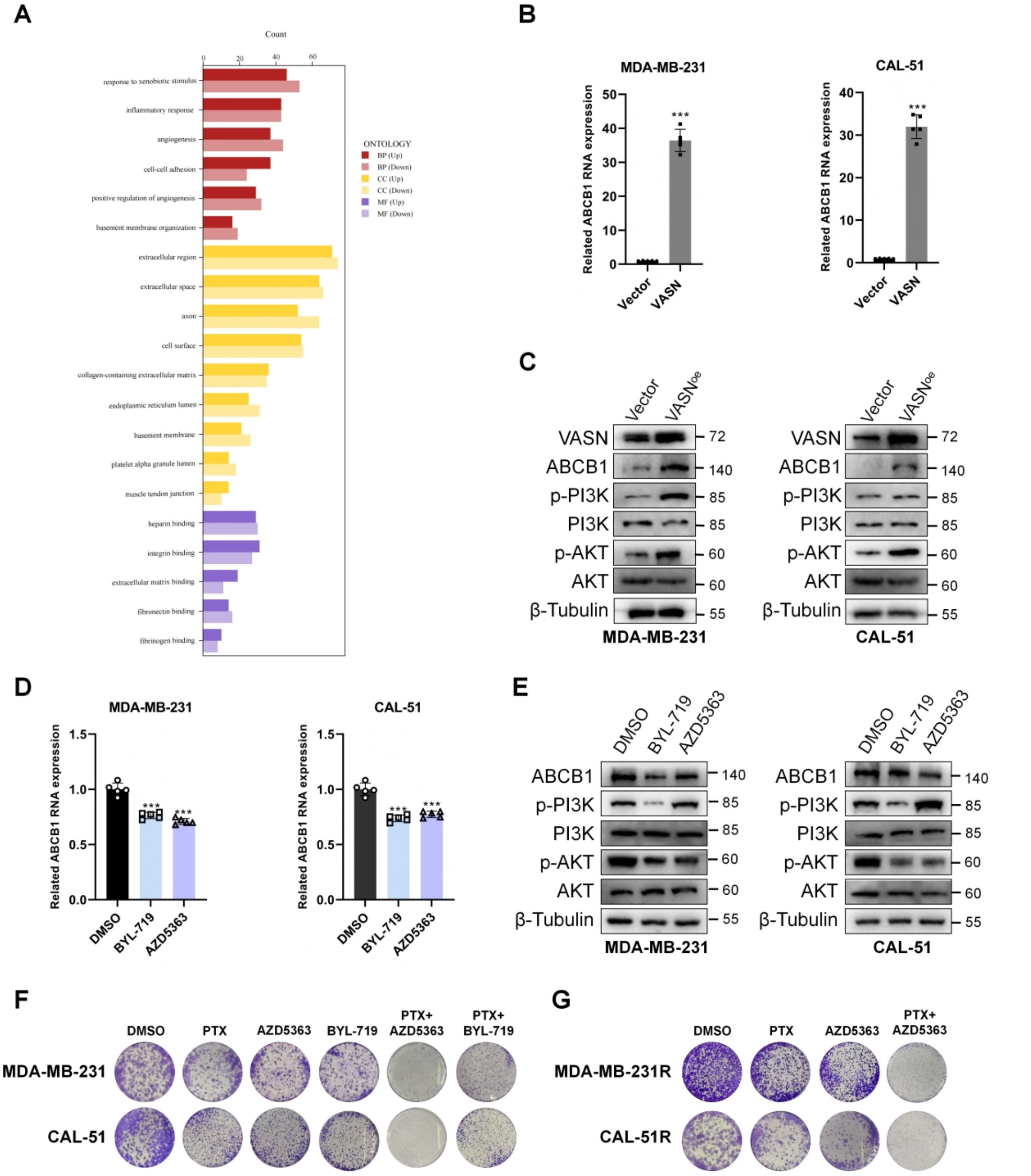

Figure S4. Overexpression of VASN activates the PI3K/AKT pathway and upregulates the expression level of ABCB1.

(A)GO enrichment analysis of DEGs from VASN-overexpressing MDA-MB-231 cells. **(B)** qRT-PCR analysis of ABCB1 mRNA expression in VASN-overexpressing MDA-MB-231 and CAL-51 cells. ***P < 0.001. **(C)** Western blot analysis of VASN, ABCB1, p-PI3K, PI3K, p-AKT, and AKT protein levels in VASN-overexpressing MDA-MB-231 and CAL-51 cells. β-Tubulin serves as a loading control. **(D)** qRT-PCR analysis of ABCB1 mRNA expression in MDA-MB-231 and CAL-51 cells treated with PI3K inhibitor (BYL-719) or AKT inhibitor (AZD5363). ***P < 0.001. **(E)** Western blot analysis of ABCB1, p-PI3K, PI3K, p-AKT, and AKT protein levels in MDA-MB-231 and CAL-51 cells treated with BYL-719 or AZD5363. β-Tubulin serves as a loading control. **(F)** Representative colony formation images of parental MDA-MB-231 and CAL-51 cells treated with DMSO, paclitaxel (PTX) alone, PTX combined with AZD5363, or PTX combined with BYL-719. **(G)** Representative colony formation images of paclitaxel-resistant

MDA-MB-231R and CAL-51R cells treated with DMSO, PTX alone, or PTX combined with AZD5363.

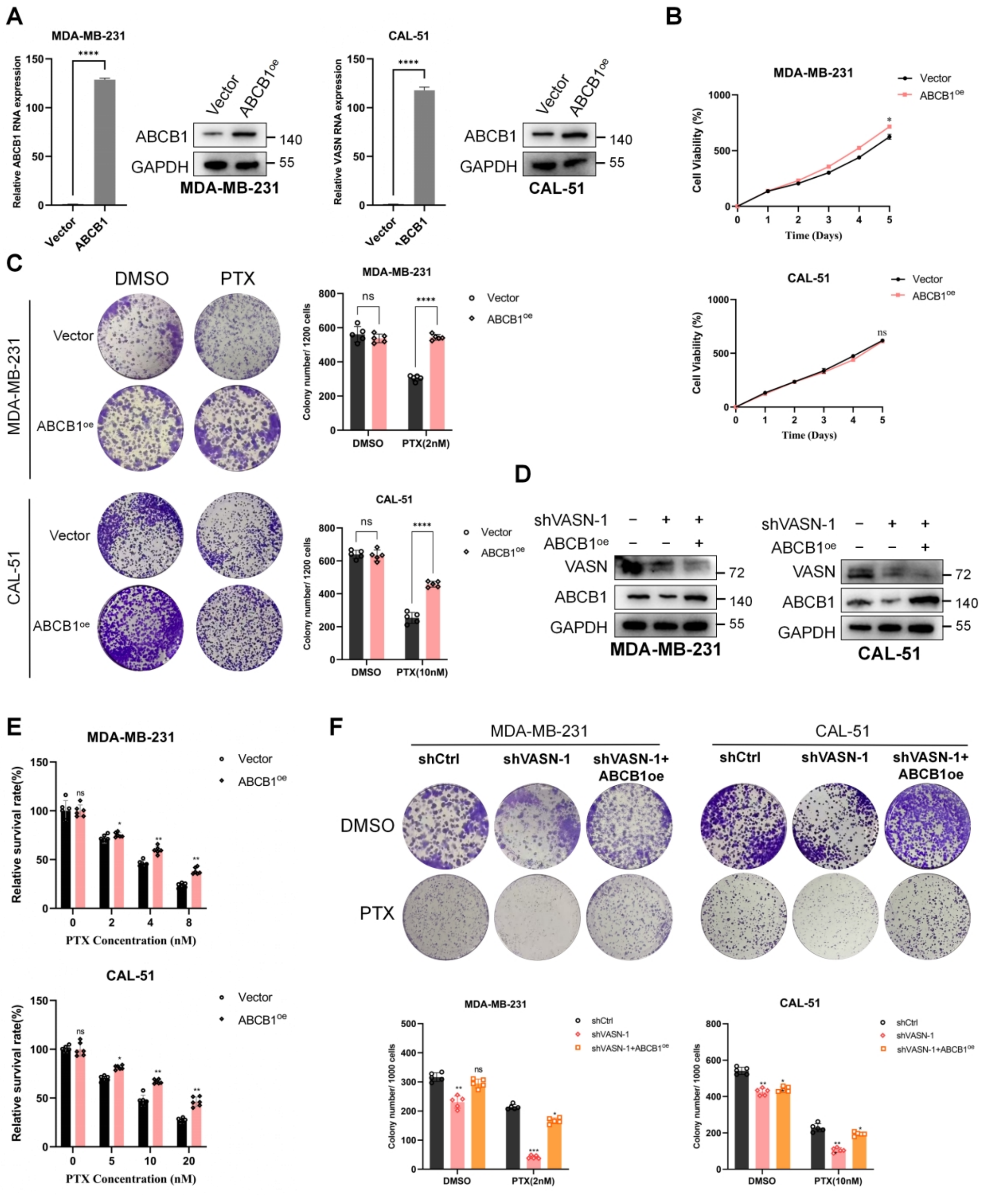

Figure S5. ABCB1 is a key downstream drug resistance gene in VASN

(A) Validation of ABCB1 overexpression efficiency in MDA-MB-231 and CAL-51 cells transfected with empty vector or ABCB1-overexpressing lentivirus, assessed by qRT-PCR (top) and Western blot (bottom). GAPDH served as a loading control. ***P < 0.001.**(B)** Cell proliferation curves of vector-control and ABCB1-overexpressing MDA-MB-231 and CAL-51 cells over 5 days. **(C)** Representative colony formation images (left) and quantification (right) of vector-control and ABCB1-overexpressing TNBC cells treated with DMSO or paclitaxel. ***P < 0.001.**(D)** Western blot analysis of VASN and ABCB1 protein expression in VASN-knockdown MDA-MB-231 and CAL-51 cells with or without ABCB1 overexpression (shVASN-1+ABCB1oe). GAPDH was used as a loading control.**(E)** Relative survival rates of VASN-knockdown MDA-MB-231 and CAL-51 cells with or without ABCB1 overexpression, treated with increasing concentrations of paclitaxel.**(F)** Representative colony formation images (top) and quantification

(bottom) of VASN-knockdown TNBC cells with or without ABCB1 overexpression, treated with DMSO or paclitaxel (2nM for MDA-MB-231, 10nM for CAL-51). ***P < 0.001.

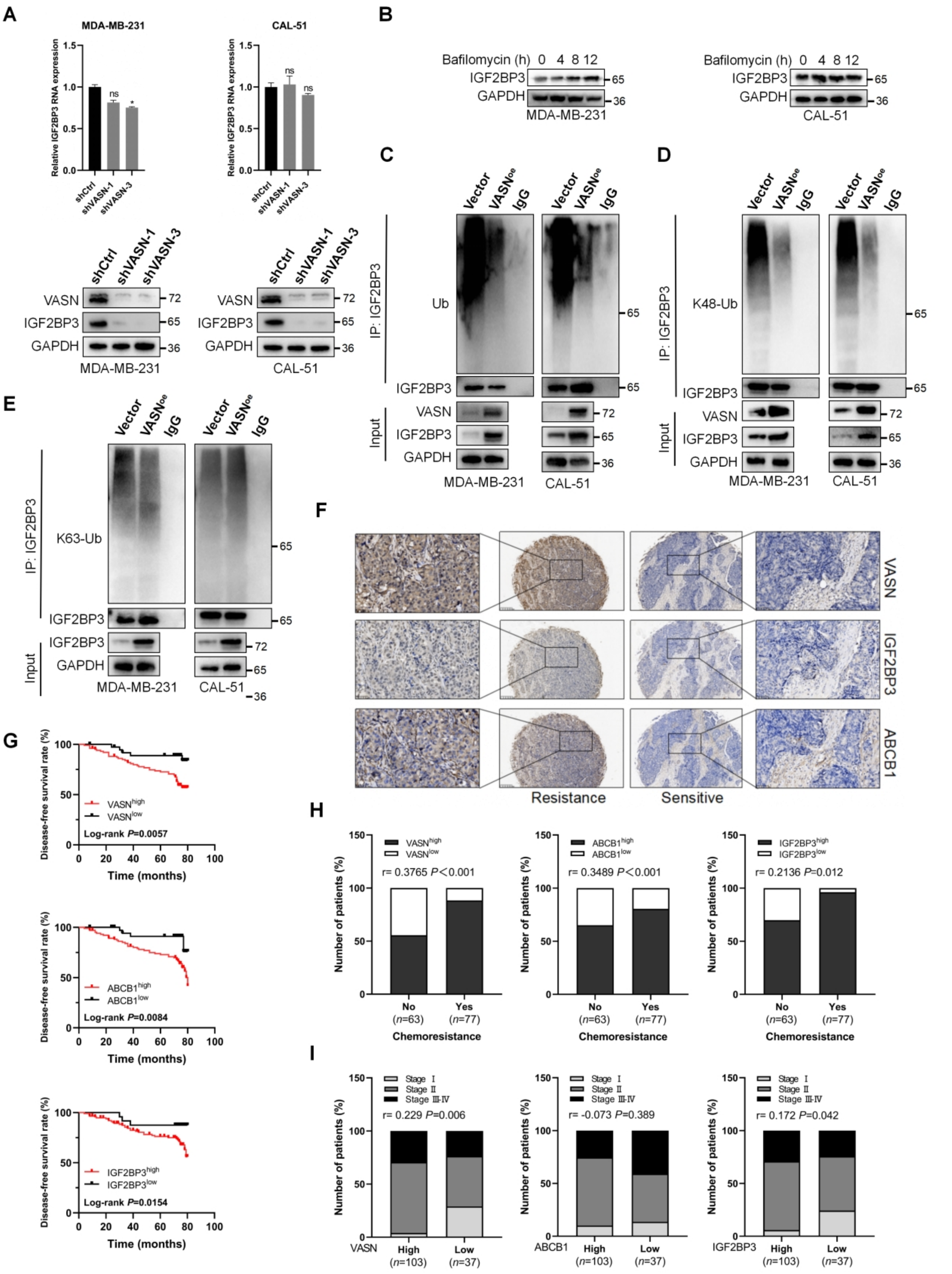

Figure S6. VASN stabilizes IGF2BP3 via K48-linked ubiquitination and correlates with poor clinical outcomes in TNBC

(A)qRT-PCR and Western blot detect the mRNA and protein levels of IGF2BP3 in VASN-knockdown MDA-MB-231 and CAL-51 cells. ns, not significant. **(B)** Western blot analysis demonstrated that lysosome inhibitor bafilomycin A1 treatment did not restore IGF2BP3 protein levels in shVASN cells. **(C, D, E)** Ubiquitination-specific IP assays confirmed that VASN overexpression (VASN^{oe}) reduced total ubiquitination and K48-linked polyubiquitination of IGF2BP3, while K63-linked polyubiquitination remained unchanged in MDA-MB-231 and

CAL-51 cells. **(F)** Representative IHC images of VASN, IGF2BP3, and ABCB1 expression in TNBC patient tissues. **(G)** Kaplan–Meier disease-free survival (DFS) curves for TNBC patients stratified by VASN, ABCB1, or IGF2BP3 expression (high vs. low). Log-rank P-values are indicated. **(H)** Bar charts showing the correlation between high expression of VASN, ABCB1, or IGF2BP3 and chemoresistance in TNBC patients. Correlation coefficients (r) and P-values are provided. **(I)** Bar charts showing the correlation between high expression of VASN, ABCB1, or IGF2BP3 and advanced tumor stage in TNBC patients. Correlation coefficients (r) and P-values are provided.

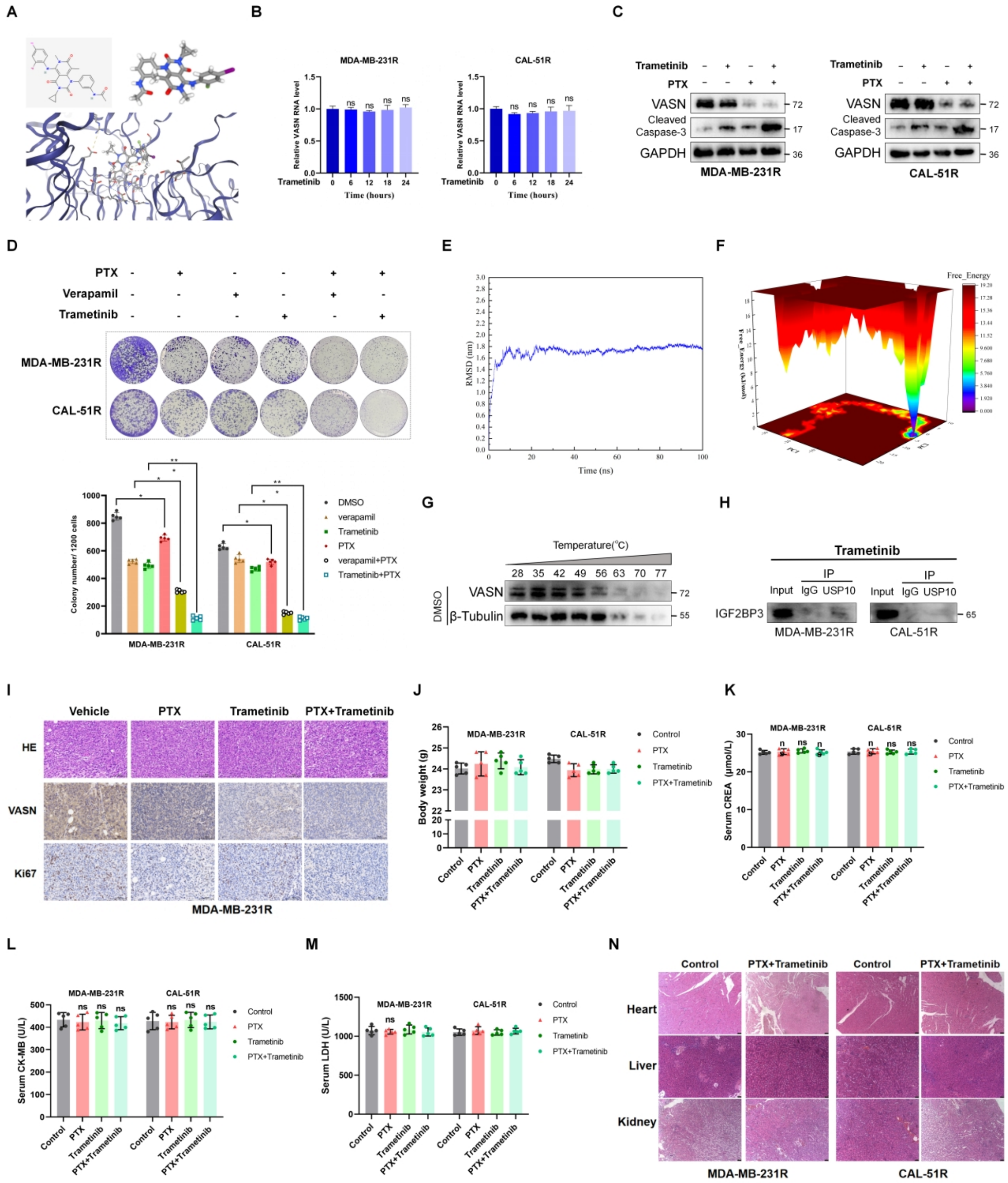

Figure S7. Trametinib combined with paclitaxel shows favorable biosafety in TNBC models

(A) Chemical structure of Trametinib and its molecular docking model with VASN, showing stable binding via hydrogen bonds and hydrophobic interactions. **(B)** qRT-PCR analysis showing that Trametinib treatment (2 μM, 0–24 h) did not significantly alter VASN mRNA expression in MDA-MB-231R and CAL-51R cells. ns, not significant. **(C)** Western blotting was performed to detect the expression of VASN, cleaved caspase-3 in two paclitaxel-resistant cells (MDA-MB-231R and CAL-51R). **(D)** Colony formation of MDA-MB-231R and CAL-51R cells treated with DMSO, paclitaxel, verapamil, Trametinib, or PTX+Trametinib. **(E)** Root-mean-square deviation (RMSD) of the VASN protein backbone atoms relative to the initial structure over 100 ns of simulation. **(F)** Free energy landscape (FEL) constructed from principal

component analysis (PCA), showing the distribution of conformational states and the thermodynamically most stable basin. **(G)** Western blot analysis of VASN protein levels in cells treated with DMSO at various temperatures(28–77 °C). β-Tubulin was used as a loading control. **(H)** The interaction between USP10 and IGF2BP3 in paclitaxel-resistant TNBC cells after Trametinib treatment. **(I)** Representative IHC staining of VASN and Ki-67 in MDA-MB-231R xenograft tumors from mice treated with vehicle, PTX, Trametinib, or PTX+Trametinib. **(J)** Body weight of mice bearing MDA-MB-231R or CAL-51R xenografts during treatment is shown (ns, $P > 0.05$). **(K-M)** Serum levels of creatinine (CREA)**(K)**, creatine kinase-MB (CK-MB)**(L)**, and lactate dehydrogenase (LDH)**(M)** in MDA-MB-231R and CAL-51R xenograft mice, indicating no significant organ damage in any treatment group (ns, $P > 0.05$). **(N)** Representative H&E staining of heart, liver, and kidney tissues from MDA-MB-231R and CAL-51R xenograft mice treated with control or PTX+Trametinib, showing well-preserved histological structures.

Supplementary Table 1. Cell sources.

Cell line	Source
MCF10A	ATCC CRL-10317
T47D	ATCC HTB-133
MCF7	ATCC HTB-22
BT474	ATCC HTB-20
MDA-MB-231	ATCC HTB-26
SKBR3	ATCC HTB-30
CAL-51	DSMZ ACC-302

Supplementary Table 2. The relationship between VASN expression and clinical features in TNBC patients with NACT

Characteristics	All cases	VASN		Chi-square value	*P* value
		Low	High		
All cases	140	36	104		
Age				1.731	0.188
<50	57	18	39		
≥50	83	18	65		
Menopausal status				2.949	0.086
Premenopausal	46	16	30		
Postmenopausal	94	20	74		
T stage				3.587	0.058
T1/T2	41	15	26		
T3/T4	99	21	78		
N stage				11.816	0.003*
N0	81	29	52		
NX	59	6	53		
TNM stage				29.327	＜0.001*
I/II	59	29	30		
III/IV	81	7	74		
Histological grading				4.599	0.032*
Grade 1/2	39	15	24		
Grade 3	101	21	80		
KI67 index (%)				7.037	0.008*
≤50	52	20	32		
＞50	88	16	72		
NACT outcome				17.622	＜0.001*
pCR	63	27	36		
Non-pCR	77	9	68		

*NACT neoadjuvant chemotherapy, TNBC Triple-negative breast cancer

* *P* values were determined by chi-square tests. Bold values indicate statistical significance ($P < 0.05$)

Supplementary Table 3. Primers used in the study.

Gene name	Primer	Sequence
VASN	Forward (5’ to 3’)	CCACCTGCCCTTTGTCCTG
	Reverse (5’ to 3’)	CAACCTGCCGCTCCTCATT
CEBPB	Forward (5’ to 3’)	GCGGGAACGCAACAGTACC
	Reverse (5’ to 3’)	TCAGCTGCTCCGACTCCTT
ABCB1	Forward (5’ to 3’)	CCCATCATTGCAATAGCAGG
	Reverse (5’ to 3’)	GTTCAAACTTCTGCTCCTGA
ALDOA	Forward (5’ to 3’)	AGCAGCAGCAGCAGCAGC
	Reverse (5’ to 3’)	TCAGTCAGTCAGTCAGTCAG
USP10	Forward (5’ to 3’)	CTGAAGCCGTTGAAAAAGATGAG
	Reverse (5’ to 3’)	TCAGCCTCTGCGTTAGAGTTG
GAPDH	Forward (5’ to 3’)	CAATGACCCCTTCATTGACC
	Reverse (5’ to 3’)	GACAAGCTTCCCGTTCTCAG

Supplementary Table 4. The sequences of shRNAs or siRNAs.

Identifier	Forward (5'-3')
shCUED1#1	GGCTGCCAGTGCAGCCAGCCACA
shCUED1#2	CCAGCCTGGTGCCTTCGACACGC
shCUED1#3	CCTGGCTGCCCTGCAGGAGCTGG
shCtrl	TTCTCCGAACGTGTCACGT
siCEBPB#1	GCAUGAGCGCCGACUACAAdTdT
siCEBPB#2	CCAACAAGGUGCUGAACUAdTdT
siABCB1#1	GGAUGAAUUCCAGAAUGAAdTdT
siABCB1#2	GCUAGUGAUUCCCAUACUAdTdT
siIGF2BP3#1	GCUAUGAUGAUGAUGAAGAdTdT
siIGF2BP3#2	GGAUGCAGAUCAUCCUGAAdTdT
shUSP10	GCCTGATCTATGAGAAGAA
siMETTL3	GCACUUGGAUCUACGGAAUdTdT
siMETTL14	GCAUGAAGAGCUGAAGAAUdTdT
siCtrl	UUCUCCGAACGUGUCACGUdTdT

Supplemental Table 5. The specific primers for m6A RIP-qPCR

Primers	Primer sequences
ABCB1	F: 5'-CCAAAATTTCACGTCTTGGTG-3'
	R: 5'-AAGTTCTTCTTCTTTGCTCCTC-3'
EEF1A positive	F:5'-CGGTCTCAGAACTGTTTGTTTC-3'
	R:5'-AAACCAAAGTGGTCCACAAA-3'
EEF1A negative	F:5'-GGATGGAAAGTCACCCGTAAG-3'
	R:5'-TTGTCAGTTGGACGAGTTGG-3'

Supplemental Table 6. The clinical and pathological information of patients with transcriptome sequencing in this study

Group	Title	Age	T staging	N staging	Histological grading	Ki-67 index
Sensitive	T1	46	T2	N1	III	55
	T2	52	T2	N2	II	45
	T3	38	T3	N1	III	30
	T4	55	T2	N0	II	35
	T5	43	T3	N1	III	20
	T6	48	T2	N0	III	30
	T7	43	T2	N1	II	40
Resistant	T8	52	T2	N0	II	25
	T9	51	T2	N0	III	25
	T10	48	T2	N2	III	35
	T11	43	T2	N0	II	20
	T12	46	T2	N1	III	25
	T13	39	T3	N0	II	55
	T14	42	T2	N1	III	30
	T15	50	T2	N1	II	40

Supplemental Table 7. Primer sequences used for PCR analysis in ChIP assay

Primers	Primer sequences
ChIP primer1	F: 5’-GCTCTGAGCCCTTGTTGCTA-3’
	R: 5’-AGCCAGGAAACAGGGAAGAG-3’
ChIP primer2	F:5’-CCAACCTGTTTCCCTCTTCC-3’
	R:5’-GCGAGAAGGTGAAGGTGAAG-3’

Supplemental Table 8. Univariate and multivariate regression analyses of achieving pCR in TNBC patients

Characteristics	Univariate analysis HR (95% CI)			*P* value	Multivariate analysis HR (95% CI)			*P* value
	HR	5% CI	95% CI		HR	5% CI	95% CI	
Age								
＞45 vs ≤45	0.875	0.527	1.452	0.502				
Menopausal status								
Postmenopausal vs Premenopausal	0.668	0.386	1.162	0.296				
NLR index								
＞1.96 vs ≤1.96	1.706	1.024	2.882	**0.045**	1.327	0.726	2.425	0.358
MLR index								
＞0.20 vs ≤0.20	1.854	1.102	3.120	**0.026**	1.606	0.870	2.966	0.130
T_stage								
T1	1 (reference)							
T2	0.588	0.264	1.244	0.092				
T3/T4	0.403	0.192	1.008	0.068				
N_stage								
N0	1 (reference)				1 (reference)			
N1	0.764	0.425	1.275	0.312	0.866	0.583	1.425	0.441
N2/N3	0.432	0.242	0.894	**0.028**	0.556	0.378	0.922	**0.036**
Histological grading								
III vs I/II	1.407	0.785	2.522	0.252				
KI67 index (%)								
≤ 20	1 (reference)				1 (reference)			
(20, 50]	1.976	1.112	3.674	**0.024**	2.179	1.141	4.164	**0.018**
＞50	8.542	3.884	18.622	**0.001**	12.038	5.054	28.674	**＜0.001**
P53 status								
Positive vs Negative	1.201	0.686	2.101	0.522				
VASN expression level								
High vs Low	0.552	0.382	0.848	**0.004**	0.583	0.412	0.882	**0.012**

BMI Body Mass Index, **ER** Estrogen Receptor, **PgR** Progesterone Receptor, **NLR** Neutrophil-to-Lymphocyte Ratio, **MLR** Monocyte-to-Lymphocyte Ratio, **HR** Hazard Ratio, **CI** Confidence Interval
